# High-resolution ribosome profiling reveals translational selectivity for transcripts in bovine preimplantation embryo development

**DOI:** 10.1242/dev.200819

**Published:** 2022-11-03

**Authors:** Linkai Zhu, Tong Zhou, Rajan Iyyappan, Hao Ming, Michal Dvoran, Yinjuan Wang, Qi Chen, R. Michael Roberts, Andrej Susor, Zongliang Jiang

**Affiliations:** ^1^ School of Animal Sciences, AgCenter, Louisiana State University, Baton Rouge, LA 70803, USA; ^2^ Department of Physiology and Cell Biology, University of Nevada, Reno School of Medicine, Reno, NV 89557-0352, USA; ^3^ Laboratory of Biochemistry and Molecular Biology of Germ Cells, Institute of Animal Physiology and Genetics, CAS, 277 21 Liběchov, Czech Republic; ^4^ Division of Biomedical Sciences, School of Medicine, University of California, Riverside, CA 92521, USA; ^5^ Department of Animal Sciences, Bond Life Sciences Center, University of Missouri, Columbia, MO 65211-7310, USA

**Keywords:** Ribosome profiling, Translational selectivity, Translation, Transcription, Preimplantation embryo development, Bovine

## Abstract

High-resolution ribosome fractionation and low-input ribosome profiling of bovine oocytes and preimplantation embryos has enabled us to define the translational landscapes of early embryo development at an unprecedented level. We analyzed the transcriptome and the polysome- and non-polysome-bound RNA profiles of bovine oocytes (germinal vesicle and metaphase II stages) and early embryos at the two-cell, eight-cell, morula and blastocyst stages, and revealed four modes of translational selectivity: (1) selective translation of non-abundant mRNAs; (2) active, but modest translation of a selection of highly expressed mRNAs; (3) translationally suppressed abundant to moderately abundant mRNAs; and (4) mRNAs associated specifically with monosomes. A strong translational selection of low-abundance transcripts involved in metabolic pathways and lysosomes was found throughout bovine embryonic development. Notably, genes involved in mitochondrial function were prioritized for translation. We found that translation largely reflected transcription in oocytes and two-cell embryos, but observed a marked shift in the translational control in eight-cell embryos that was associated with the main phase of embryonic genome activation. Subsequently, transcription and translation become more synchronized in morulae and blastocysts. Taken together, these data reveal a unique spatiotemporal translational regulation that accompanies bovine preimplantation development.

## Teaser

**Summary:** High-resolution ribosome profiling of bovine oocytes and early embryos characterizes the translational landscapes of preimplantation embryo development and reveals four modes of translational selectivity for transcripts in oocytes and embryos.

## INTRODUCTION

Preimplantation embryo development is a complex and precisely regulated process orchestrated by both maternal stored mRNAs and newly synthesized transcripts that appear following embryonic genome activation (EGA). In the last decade, transcriptome analyses of early mammalian embryos from multiple species have been comprehensively conducted and have established precise gene transcription programs during preimplantation development. However, the levels of mRNA and the amount of its protein product often do not directly correlate (Becker et al., 2018), suggesting that the mRNAs detected from global transcriptomic profile do not necessarily represent their functional status in early embryo development. Although the protein expression landscape of oocytes and preimplantation embryos has been characterized in mouse (Gao et al., 2017; Wang et al., 2010) and bovine (Banliat et al., 2021, 2022; Demant et al., 2015; Deutsch et al., 2014; Marei et al., 2019), the proteomic analysis offers limited coverage and information due to scarcity of the sample material available and has not been explored in other mammalian species. More importantly, a central gap in our understanding of post-transcriptional regulation exists, namely, how mRNAs are selected for spatial and temporal regulation during cell-fate specification and in processes such as oocyte maturation, fertilization, EGA and early differentiation. Thus, the understanding of mRNA translational dynamics may provide new insights into gene regulation during embryogenesis.

Accordingly, in some systems, ribosome profiling coupled to RNA sequencing (Ribo-seq) has been developed to quantify ribosome occupancy and to analyze selective genome-wide mRNA translation (Chassé et al., 2017; Ingolia et al., 2009). However, the broad application of Ribo-seq has been slowed by its complexity and the difficulty of adapting it to low amounts of input material. Recently, two powerful single-cell Ribo-seq (scRibo-seq) protocols have been developed. The first, Ribo-STAMP (Surveying Targets by APOBEC-Mediated Profiling), utilizes a cytosine deaminase (APOBEC) that catalyzes RNA cytosine-to-uracil conversion to edit transcripts associated with ribosomes (Brannan et al., 2021). The second scRibo-seq protocol utilizes the micrococcal nuclease MNase to digest RNA not bound to ribosomes in lysates of single cells and allows the capture of the ribosome-protected footprints (VanInsberghe et al., 2021). Both approaches require complex quality control and analysis due to the high ‘noise’ observed with single-cell data. In addition, it has been shown that mRNAs engaged in translation are bound by ribosomes, whereas dormant or stored transcripts are accumulated in diverse forms of ribonucleoprotein complexes and particles (Anderson and Kedersha, 2006; Eulalio et al., 2007; Parker and Sheth, 2007). It is also well known that actively translated mRNAs are bound by multiple ribosomes, or polysomes. The above-mentioned approaches limit analysis of the variation encountered in the different numbers of ribosome-bound mRNAs as a whole, while ignoring how the specific mRNAs are preferentially selected for translation. It should be noted that two recent studies have also optimized a low-input ribosome profiling (LiRibo-seq) approach and provided for the first time the translational dynamics of mouse oocytes and preimplantation embryos (Xiong et al., 2022; Zhang et al., 2022), but again, these two studies were confined to an analysis of ribosome-bound mRNAs as a whole. In contrast, an imaging-based approach performed on living *Drosophila* embryos has allowed the direct exploration of the location and dynamics of translation of individual mRNAs (Dufourt et al., 2021), and has opened up new avenues for understanding gene regulation during development; however, this technology is still in its infancy.

In our study, we have improved a recent advance of scarce sample polysome profiling (SSP-profiling) (Masek et al., 2020) based on physical polysome fractionation (Chassé et al., 2017; Scantland et al., 2011). We substantially increased the resolution of the procedure to enable the sequencing of transcripts associated with monosomes and different sizes of polysomes extracted from bovine oocytes and preimplantation embryos. The data obtained have allowed us to study both genome-wide translational dynamics and translational selectivity mechanisms that accompany bovine early embryo development.

## RESULTS

### mRNA translational landscapes in bovine oocytes and preimplantation embryos

**Fig. 1.**
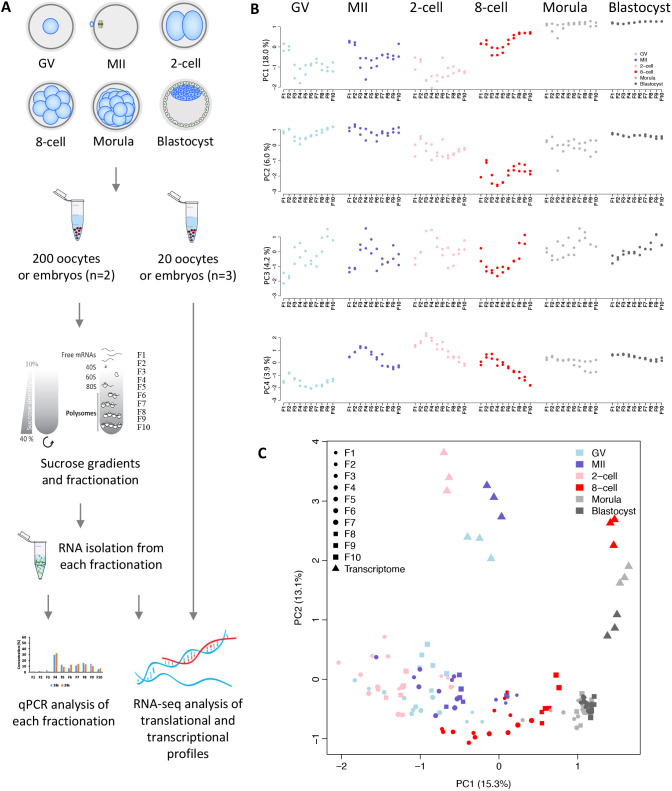
**Genome-wide high-resolution ribosome profiling of bovine oocytes and early embryos.** (A) Scheme of genome-wide high-resolution polysome profiling in bovine oocytes and preimplantation embryos. (B) Principal component analysis (PCA) of polysome- and nonpolysome-bound mRNA profiles in ten fractions of bovine oocytes and early embryos. (C) PCA analysis of translatomes (F1-F10) (*n*=2) and transcriptomes (*n*=3) of bovine oocytes and early embryos.

**Fig. 2.**
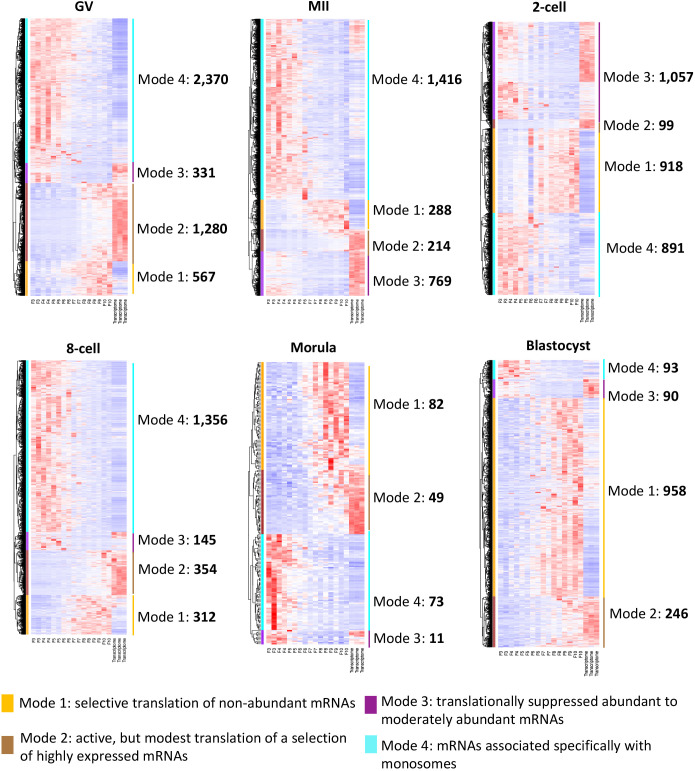
**Diverse modes of translational selectivity during bovine oocyte and preimplantation development.** Heatmaps showing four modes of translational selectivity in bovine oocyte and preimplantation development. The color spectrum, ranging from red to white to blue, indicates high to low levels of gene expression. Mode 1, selective translation of non-abundant mRNAs (gold bar); mode 2, active, but modest translation of a selection of highly expressed mRNAs (brown bar); mode 3, translationally suppressed abundant to moderately abundant mRNAs (purple bar); and mode 4, mRNAs associated specifically with monosomes (cyan bar). The numbers of genes identified in individual modes of in each development stage are indicated.

Polysome profiling has traditionally required a large amount of input material in order to fractionate polysome-bound RNA, making the procedure challenging to apply to mammalian oocytes and embryos. SSP-profiling (fractionation of mRNAs based on the number of translating ribosomes by using sucrose-density gradients) overcomes some of the obstacles posed by low sample size (Masek et al., 2020). The improved SSP-profiling when followed by RNA sequencing (RNA-seq) allowed us to analyze mRNA translational profiles of bovine oocytes at the germinal vesicle (GV) and metaphase II (MII) stages, as well as of preimplantation embryos at the two-cell, eight-cell, morula and blastocyst stages (Fig. 1A). For each sample, 100 oocytes or embryos were used, and the experiment was performed twice. We split each developmental stage by ultracentrifugation on sucrose gradients into ten equal volumes of fractions to provide a high resolution translatomic profile (transcripts associated with ribosomes from all fractionations) (Fig. 1A). We conducted two analyses to validate the translatomic data. First, we assessed the RNA isolated from each of the ten fractions by quantitative reverse transcription PCR (qRT-PCR)-based quantification of 18S and 28S ribosomal RNA (rRNA) (Fig. S1), which allowed us to confirm the successful separation of free RNAs, 40S small ribosomal subunits, 60S large ribosomal subunits, monosomes (80S) and polysomes (see Materials and Methods). The amount of 18S and 28S RNA provided an assessment of the reproducibility of fraction collection (Fig. S1). Additionally, principal component (PC) analysis (PCA) and Pearson correlation analysis of translatomic data indicated consistent values between biological replicates in each fraction and across developmental stage (Fig. 1B, Fig. 2). Based on these analyses, we classified the ten fractionations into free RNA (F1-F2), monosome-bound mRNA (F3-F5, with F6-F7 as an intermediate stage) and polysome-bound mRNA (F8-10, regarded as polysomes hereafter) profiles. In addition to ribosome-profiling analysis, global transcriptome analysis was performed on 20 oocytes (GV and MII stages) (*n*=3) and 20 embryos (*n*=3) at each developmental stage collected from the same batches used for ribosome fractionation and RNA-seq profiling. The transcriptomic data (triangles in Fig. 1C), especially in the PC1 dimension, appeared to organize roughly as two, seemingly distinct groupings, namely, the stages representing oocytes and two-cell-stage embryos and the stages from eight-cell to blastocyst (Fig. 1C), which is consistent with the notion that bovine major EGA occurs at the eight-cell stage (Graf et al., 2014; Jiang et al., 2014).

Overall, the translatome profile contrasted markedly with the transcriptome profile across different development stages (Fig. 1C), suggesting discordance between the global transcriptome and actively translated mRNAs. Again, there was a separation of the translatome data by stage. In particular, the morula and blastocyst values were clustered together at the far right of the PC1 plot and well distanced from early-stage data, which were clustered mainly towards the left of the PC1 plot and further separated from the rest of the developmental stages. Values for the eight-cell embryos fell somewhere in between (Fig. 1B,C). Our data also indicated that the changes in the translatome appeared to be gradual across the fractions from F1 to F10 (Fig. 1B,C), reflecting the continuous physical fractionation of mRNAs based on the number of translating ribosomes. Although considerable differences existed between the transcripts that were transcribed and those that were translated, the various PCAs confirmed the largely similar trajectories of translatome and transcriptome dynamics during the development transition from oocytes to blastocysts, with a major shift occurring at the crucial eight-cell stage (Fig. 1C).

### Diverse modes of translational selectivity during bovine oocyte and preimplantation development

To delineate the relationship between translation and transcription during bovine oocyte and preimplantation development, we assessed the correlation of all the detected genes between the translatome and the transcriptome that had been generated from each of the six developmental stages. The F1-F2 fractions were excluded in order to allow us to focus on the translatome analysis (see Materials and Methods). Overall, we found considerable differences between polysome-occupied (F8-10) and monosome-occupied (F3-F5) mRNAs over the course of development (Fig. 2). We identified four modes of translational selectivity in each developmental stage: mode 1, selective translation of non-abundant mRNAs (Fig. 2, gold bar); mode 2, active, but modest translation of a selection of highly expressed mRNAs (Fig. 2, brown bar); mode 3, translationally suppressed abundant to moderately abundant mRNAs (Fig. 2, purple bar); and mode 4, mRNAs associated specifically with monosomes (Fig. 2, cyan bar). A complete list of genes (Fig. 2) from the four identified modes across bovine oocyte and preimplantation development are presented in Table S1, which should provide a valuable resource for others interested in translational regulation during bovine early embryo development.

Analysis of the functions of genes in mode 2 (active, but modest translation of a selection of highly expressed mRNAs) revealed a sequential progression of stage-specific gene networks. Gene enrichments shifted from ‘cell division’, ‘chromosome organization’ and ‘mitotic nuclear division’ in oocytes (GV and MII stages), to ‘embryonic cleavage’ and ‘regulation of DNA replication’ in two-cell embryos, to ‘translation’ in eight-cell embryos, and finally to ‘cell-cell adhesion’ and ‘protein folding’ in the morula and blastocyst stages, when junctional complexes between cells become evident (Table 1).

**Table 1.**
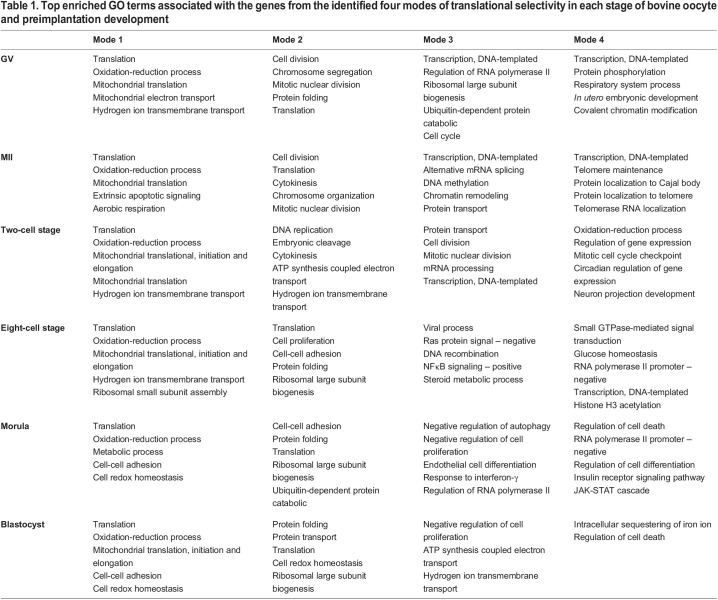
Top enriched GO terms associated with the genes from the identified four modes of translational selectivity in each stage of bovine oocyte and preimplantation development

Besides the gene groups that were highly expressed and actively translated, we identified a second class of genes, sometimes relatively large in number, which had a low abundance of transcripts; however, these transcripts appeared to be actively translated as they were occupied by polysomes (mode 1, selective translation of non-abundant mRNAs; Fig. 2). The common dominant biological processes represented in this mode included ‘translation’, ‘oxidation-reduction process’ and ‘mitochondrial translation’; these functions were evident across all developmental stages (Table 1). Stage-specific programs included ‘hydrogen ion transmembrane transport’ and ‘apoptotic signaling’ from the oocyte to the eight-cell stage, and ‘cell-cell adhesion’ and ‘cell redox homeostasis’ at the morula and blastocyst stages (Table 1).

The highly expressed but poorly translated transcripts (mode 3, translationally suppressed abundant to moderately abundant mRNAs; Fig. 2) were primarily involved in ‘transcription, DNA-templated’ and ‘RNA regulation’ in oocytes, ‘protein transport’ and ‘cell division’ at the two-cell stage, ‘viral process’ and ‘Ras protein signal transduction’ at the eight-cell stage, and ‘negative regulation of autophagy’ and ‘negative regulation of cell proliferation’ at the morula and blastocyst stages (Table 1).

We also identified mRNAs occupying monosomes (mode 4) from each developmental stage (Fig. 2). Gene Ontology (GO) analysis indicated significant gene enrichments related to ‘transcription, DNA-templated’ and ‘protein phosphorylation’ at the GV stage, ‘transcription, DNA-templated’ and ‘telomerase protein localization’ at MII, ‘oxidation-reduction process’ and ‘regulation of gene expression’ at the two-cell stage, ‘small GTP signal transduction’ and ‘glucose homeostasis’ at the eight-cell stage, ‘regulation of cell death’ and ‘cell differentiation’ at the morula stage, and ‘intracellular sequestering of iron ion’ and ‘regulation of cell death’ at the blastocyst stage (Table 1).

We then sought to understand how such modes of translational selectivity are established. First, we performed a genome-wide correlation between the transcripts that constituted the four different modes and certain characteristic mRNA features. These features included the presence of cytoplasmic polyadenylation elements (CPEs), known to be important for translational regulation (Piqué et al., 2008), and 3′ untranslated regions (UTR) and 5′ UTR lengths. We observed that the transcripts in mode 1 [highest translational efficiency (TE) of polysome/mRNA] had the lowest CPE number and density, whereas transcripts in mode 2 (moderate TE) and mode 3 (lowest TE) demonstrated a higher CPE number and density than those of mode 1 both before (Fig. S2A) and after the EGA stage (Fig. S2B). When the TE was compared with CPE number and density on all detected transcripts, we confirmed that these values were negatively correlated (Fig. S2C,D).

The decrease in TE in the progression from mode 1 to mode 4 was also accompanied by increased lengths of 3′ UTRs, but not of 5′ UTRs of the transcripts (Fig. S3A,B) across all stages, and, for all transcripts identified, TE was in general negatively correlated with 3′ UTR length and positively correlated with 5′ UTR length. It should be noted, however, that these correlations were quite weak (Fig. S2C,D). Taken together, these data reveal a role of CPEs, and possibly the lengths of the 3′ UTRs and 5′ UTRs for translational regulation in bovine early embryonic development.

Finally, we calculated the proportion of maternal or embryonic transcripts in each of the four modes across developmental stages. The proportion of maternal transcripts was high and that of embryonic transcripts low in all four modes in the early stages (GV through two-cell stage) of development (Fig. S4A,B). At the eight-cell stage and thereafter, when transcription from the embryonic genome became much more active, the proportion of embryonic transcripts, as expected, rose markedly, especially in modes 1 and 2 at the eight-cell stage (Fig. S4B). The eight-cell stage was also distinguished by a high proportion of remaining maternal transcripts occupying monosomes (Fig. S4A). By the morula stage, maternal transcripts associated with ribosomes were rare; however, we observed a high proportion of monosome-bound maternal transcripts that persisted to the blastocyst stage (Fig. S4A).

Collectively, our analysis captured four modes of translational selectivity for transcripts during bovine oocyte and preimplantation development. In particular, the analysis revealed gene activities (modes 1 and 3) that could not be readily inferred from transcriptomic data alone.

### Translational control in bovine oocyte and preimplantation development

**Fig. 3.**
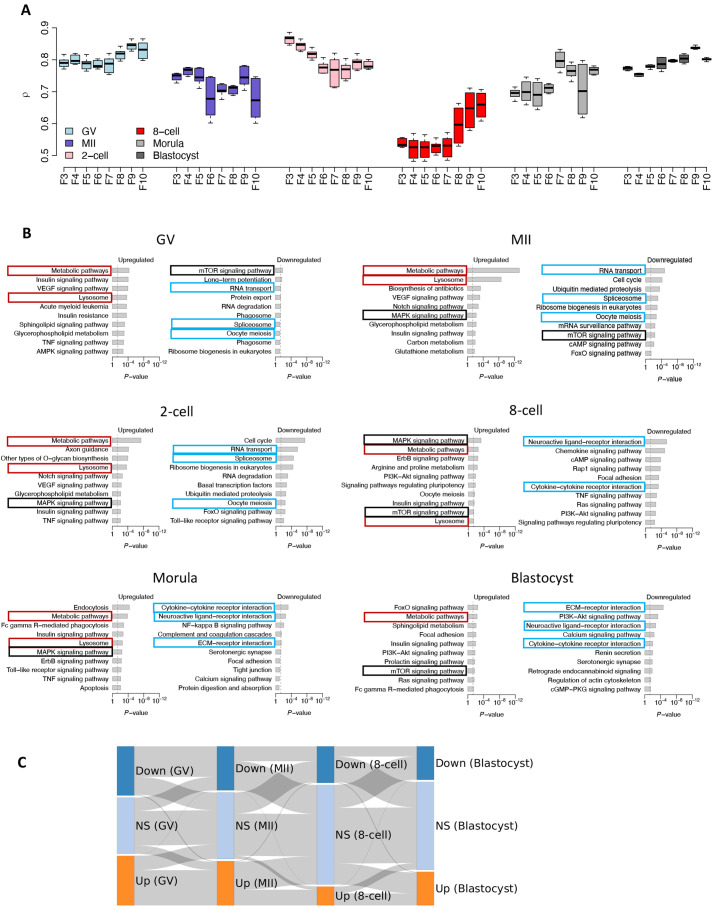
**Translational control in bovine oocyte and preimplantation embryo development.** (A) Translational control is summarized by the correlation analysis of the translatome (F3-F10) and the transcriptome at each developmental stage. Data show the mean±s.e.m. *n*=2. (B) KEGG pathway analysis of the differentially expressed genes between polysome-occupied mRNAs (F8-F10) and the transcriptome in bovine oocyte and preimplantation development. Red boxes highlight commonly upregulated pathways that are preferentially translated throughout bovine oocyte and preimplantation development. Cyan boxes highlight commonly downregulated pathways that are inactive or translated before (GV, MII and two-cell stage) or after (eight-cell, morula and blastocyst stage) major EGA stages. Black boxes highlight the most dynamic pathways that are translationally controlled throughout early development. Upregulated or downregulated pathways: FDR<0.05, FC>8; KEGG disease pathways are excluded. (C) Sankey diagram showing the upregulated and downregulated genes (FDR<0.05) between polysome-occupied mRNAs (F8-F10) and the transcriptome in each developmental stage. Down, downregulated; Up, upregulated; NS: not significantly regulated.

To gain insight into the broad translational regulation landscape across bovine oocyte and preimplantation development, we integrated the translatomes, i.e. transcripts associated with polysomes, with transcriptomes. The correlation between the translatome and the transcriptome was reasonably robust in GV and MII oocytes and in two-cell embryos, but appeared strongest in GV oocytes (Fig. 3A), in which transcription is silenced, with the oocytes relying largely on abundant maternally stored RNAs, which are translated for oocyte growth and for the oocyte maturation process (Schultz et al., 2018). Translatomic data correlated less well with the transcriptome in MII oocytes than in GV oocytes, in which there remains a reliance on maternal transcripts but with more selective translation from the embryonic genome, possibly in preparation for fertilization (Schultz et al., 2018). In contrast to the earlier stages, marked translational control was observed in eight-cell embryos (Fig. 3A). In other words, polysome occupancy poorly reflects the transcriptome, most likely because the eight-cell stage is when large-scale transcription of the embryonic genome is being initiated, but the newly synthesized mRNAs may not yet fully occupy the ribosomal machinery. Of note, partial polysome-occupied mRNAs were selected to be translated immediately in the eight-cell embryo (Fig. 3A), suggesting that these genes are essential for the major EGA. Subsequent to the eight-cell stage, translation and transcription appear to gradually become more synchronized in morulae and particularly in blastocysts (Fig. 3A), suggesting that this burst of protein production and cell proliferation is necessary to prepare the blastocyst for impending events, such as divergence of the hypoblast and epiblast.

To explore previously undefined translational dynamics in bovine oocyte and preimplantation development, we examined the pathways inferred from upregulated and downregulated, polysome-associated transcripts compared with the transcriptome at each developmental stage using a stringent cutoff with false discovery rate (FDR)<0.05 and fold change (FC)>8 (Fig. 3B). Transcripts associated with the broad term ‘metabolic pathways’ and the narrower term ‘lysosome’ were upregulated and, therefore, these mRNAs appeared to be preferentially translated throughout bovine preimplantation development (Fig. 3B). ‘RNA transport’, ‘spliceosome’ and ‘oocyte meiosis’ were pathways that were generally downregulated before the major EGA stages (GV, MII and two-cell stage), whereas commonly downregulated pathways at or after the major EGA stages (eight-cell stage, morula and blastocyst) included various ligand-receptor interactions and extracellular matrix (ECM)-receptor interactions (Fig. 3B). Additionally, classical pathways, including those for mTOR and MAPK signaling, were the most dynamic pathways translationally controlled throughout early development (Fig. 3B).

The data also revealed that the same polysome-occupied mRNAs in GV oocytes were largely retained in MII oocytes and only lost their translational selectivity at the eight-cell stage and beyond (Fig. 3C), whereas the translationally suppressed mRNAs in GV oocytes were also essentially the same as the ones identified in MII oocytes and eight-cell stage embryos (Fig. 3C).

### A translational switch occurs during bovine major EGA

**Fig. 4.**
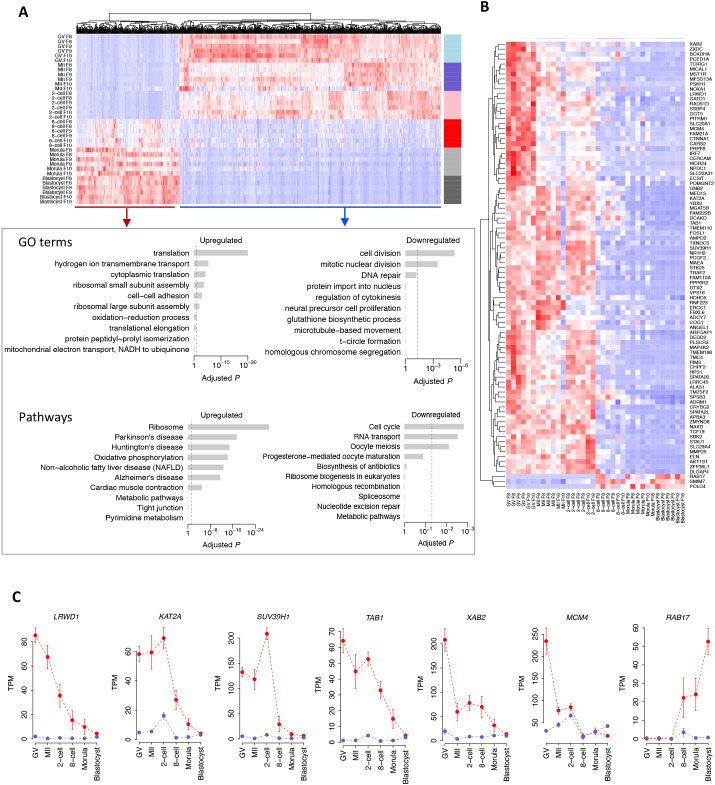
**A translational switch occurs during bovine major EGA.** (A) Heatmap (top panel) showing that the polysome-occupied mRNAs (F8-F10) are correlated with developmental progression. The color spectrum, ranging from red to white to blue, represents high to low levels of gene expression. Top enriched GO terms and KEGG pathways (bottom panel) associated with upregulated (i.e. upregulation in eight-cell, morula and blastocyst stages) or downregulated (i.e. upregulation in oocytes and two-cell embryos) polysome-occupied genes towards the developmental progression are presented. (B) Heatmap of 90 prioritized genes with the most dynamic translational selectivity across bovine oocyte and preimplantation development. The color spectrum, ranging from red to white to blue, represents high to low levels of gene expression. (C) Exemplary genes with distinct patterns between translation (red) and transcription (blue) in bovine oocyte and preimplantation development. Data show the mean±s.e.m. *n*=2 (translation), *n*=3 (transcription).

To identify the genes with distinct translational trends as development progressed, we attempted to correlate the polysome-occupied mRNAs with stage. This analysis confirmed the dramatic translatome shift associated with the major EGA stage in the eight-cell embryo (Fig. 4A, top panel). Until then, the upregulated polysome-occupied transcripts detected in the later developmental stages, i.e. the eight-cell, morula and blastocyst stages, were significantly enriched for processes associated with ‘translation’, ‘hydrogen ion transmembrane transport’, ‘cytoplasmic translation’, ‘ribosomal subunit assembly’ and ‘cell-cell adhesion’ (Fig. 4A, bottom panel), whereas pathway analysis revealed a significant enrichment for ‘ribosome assembly’ and ‘oxidative phosphorylation’ (Fig. 4A, bottom panel). The pathway analyses were also in agreement with these activities, especially in relation to energy metabolism. By contrast, the downregulated polysome-occupied transcripts from the later stages, i.e. those upregulated in oocytes and two-cell embryos, were associated with ‘cell division’, ‘mitotic nuclear division’ and ‘DNA repair’ (Fig. 4A, bottom panel), consistent with roles in oocyte maturation and the early cleavage stages. The pathway analyses were also in agreement with these activities including ‘cell cycle’, ‘RNA transport’ and ‘oocyte meiosis’, especially in relation to oocyte maturation (Fig. 4A, bottom panel).

We then identified 90 genes that have the most dynamic translational selectivity across development (Fig. 4B), of which most are actively translated in the oocyte to the two-cell stage and downregulated thereafter. Among the top ranked downregulated, polysome-occupied transcripts across developmental stages were *LRWD1*, *KAT2A*, *SUV39H1*, *TAB1*, *XAB2* and *MCM4* (Fig. 4C), all of which have functions linked to chromatin state. For example, *LRWD1* is a subunit of the origin recognition complex and plays a role in heterochromatin organization and cell cycle control (Bartke et al., 2010; Hsu et al., 2020; Wang et al., 2018a, 2017). *KAT2A* (also known as *GCN5*) is a histone acetyltransferase, whereas *SUV39H1* is a histone methyltransferase that trimethylates lysine 9 of histone H3 and plays pivotal roles in sculpting the epigenetic landscape through chromatin modification (Haque et al., 2021; Morgan and Shilatifard, 2020). Given that a hallmark feature of a competent oocyte is chromatin condensation, the surprisingly highly selective translation of these genes in oocytes (both GV and MII) and the likely role of the translated proteins in maintaining the repressive heterochromatic state suggest that, in combination, these genes may have important functions in the epigenetic control of bovine oocyte competence. *SUV39H1* and *TAB1* (Fig. 4C) have previously been shown to have essential roles in the maternal to zygotic transition (Yang et al., 2017; Zhou et al., 2021) and bovine preimplantation development (Jafarpour et al., 2020; Zhang et al., 2016, 2018), respectively. In contrast, the top-ranked upregulated polysome-occupied transcripts across developmental stages are those of *RAB17* (Fig. 4C). RAB17 belongs to a subfamily of small GTPases and plays an important role in the regulation of membrane trafficking (Lütcke et al., 1993). The translation of *RAB17*, which begins after the major EGA, is especially high at the blastocyst stage when the trophectoderm lineage emerges and the blastocoel cavity forms. Two other transcripts with similar dynamics to those of *RAB17* are *SMIM7* and *POLD4* (Fig. 4B), which encode a small integral membrane protein and a DNA polymerase subunit, respectively. However, neither appears to have anything in common with each other or with *RAB17*. Their specific functions in bovine preimplantation development are unknown.

### Genes showing discordance between transcription and translation

**Fig. 5.**
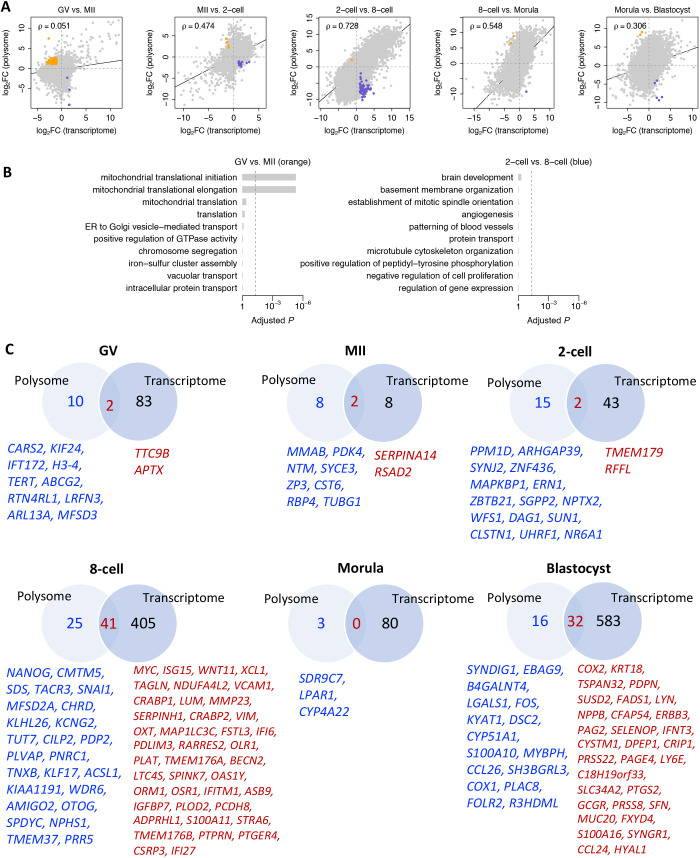
**Genes showing discordance between transcription and translation.** (A) Differential gene expression analysis between polysome-occupied mRNAs and the transcriptome in each developmental transition during bovine oocyte and preimplantation development. Gold dots represent genes that have decreased transcription but upregulation of translation in each developmental transition (FDR<0.05 and FC>2). Blue dots represent genes that have increased transcription but decreased translation in each developmental transition (FDR<0.05 and FC>2). ρ indicates Spearman correlation coefficient and the black line indicates regression. (B) The GO terms associated with the genes with decreased expression and upregulated translation in MII compared with those of genes in GV oocytes (left), and the GO terms associated with the genes with increased transcription and decreased translation during the two-cell stage and eight-cell stage transition. (C) Venn diagram showing the genes that are specifically and highly translated or transcribed in one particular stage across bovine oocyte and preimplantation development. The highly translated genes (blue) and the highly translated and most abundant genes (red) specific to each development stage are listed.

We next analyzed the genes that showed contrasting trends in transcription versus translation (FDR<0.05 and FC>2) between stages during development from the oocyte to blastocyst (Fig. 5A). Genes that had decreased transcription but an upregulation of translation are represented by gold dots, whereas genes with increased transcription but decreased translation are in blue (Fig. 5A). A total of 103 genes showed a decrease in transcript number and at the same time had increased expression in the transition from GV oocyte to the MII stage (Table S2). Annotation of these genes revealed significant enrichment of ‘mitochondrial translational initiation’ and ‘translational elongation’ (Fig. 5B). These findings suggest that oocyte maturation requires a surge in the biosynthesis of mitochondrial components, which is consistent with the reported rise in aerobic metabolism accompanying oocyte maturation and gain of oocyte competence (Wang et al., 2018b; Zhang et al., 2019a,b). By contrast, 65 genes had increased transcription but decreased translation (blue dots) during the two-cell stage and eight-cell stage transition (Table S3). However, conventional annotation analysis of these genes was not particularly informative (Fig. 5B), although it must be assumed that some of these gene products play key roles in preparation for the major EGA occurring at the culmination of this transition.

We also identified several genes that are highly translated and transcribed at one particular stage of development but have low expression at other stages (Fig. 5C), suggesting that they likely have a specific regulatory function associated with that particular transition. We used the bovine embryo proteome data that had been acquired by nanoliquid chromatography coupled with tandem mass spectrometry (Banliat et al., 2022). Transcripts for five genes (*ORM1*, *PLAT*, *SERPINH1*, *TAGLN* and *TUT7*) encoding proteins found to be abundant in eight-cell embryos were also highly expressed at this stage of development (Fig. S5). Several other genes with stage-specific expression as assessed by the number of polysome-bound transcripts (*CARS2*, *CST6*, *DAG1*, *MMAB*, *SUN1*, *TUBG1*, *UHRF1*, *WFS1* and *ZP3*) were also validated by their protein expression (Fig. S5). Finally, the well-known pluripotency genes *NANOG*, *KLF17* and *MYC* and the interferon-response gene *ISG15* were highly translated and transcribed at the eight-cell stage but much less so elsewhere. Again, the major EGA stage appears to be one that is particularly dynamic with regard to changes in gene expression.

## DISCUSSION

Early embryonic loss greatly affects fertility of both humans and agriculturally important animals such as cattle, yet the underlying causes are for the most part unknown. A characterization of the molecular events accompanying the maturation of the oocyte, fertilization and the early cleavage stages of embryonic development may provide some insight into what can potentially go wrong in the pregnancies that fail in these early stages. Omics technologies have enabled in-depth analysis of molecular mechanisms of bovine preimplantation development including a catalog of the transcripts (Graf et al., 2014; Jiang et al., 2014; Kues et al., 2008; Misirlioglu et al., 2006; Xie et al., 2010) and proteins (Banliat et al., 2021, 2022; Demant et al., 2015; Deutsch et al., 2014; Marei et al., 2019) present; the state of the epigenome, for example, DNA methylation status (Duan et al., 2019; Jiang et al., 2018); chromatin dynamics (Halstead et al., 2020; Ming et al., 2021); histone modifications (Lu et al., 2021); and the expression of small RNAs (Cuthbert et al., 2021; Cuthbert et al., 2019). However, the mRNA translation landscape and particularly the translational controls operating on specific mRNAs in oocytes and embryos remain largely unstudied. Here, we have developed a low-input, high-resolution, ribosome-profiling approach and provided a genome-wide characterization of the important but often overlooked translational regulation process. The datasets, particularly when mined in further detail and integrated with epigenome information, are expected to greatly expand our understanding of the gene regulation mechanisms governing bovine embryonic development. Perhaps most importantly, significant discordance was frequently observed to exist between the linked processes of translation and transcription at each developmental stage of bovine early development, highlighting the importance of evaluating the translatome in addition to the more accessible transcriptome. Our study represents the first insights into mRNA translational dynamics and a comparison of the transcriptome with polysome- and non-polysome-bound mRNA profiles during mammalian oocyte and preimplantation development. In this regard, the bovine is recognized as a highly informative model for human embryo development (Daigneault et al., 2018; Halstead et al., 2020; Jiang et al., 2014; Rossant, 2011), on which such experiments are profoundly more difficult to conduct.

Our study was able to capture four diverse, although somewhat empirical, modes of translational selectivity for transcripts. In particular, mode 1 (selective translation of non-abundant mRNAs) and mode 3 (translational suppression of abundant to moderately abundant mRNAs) provide information that could not be inferred by transcriptome analysis alone. The mRNAs in mode 1 provide a database for transcripts that are prioritized for translation relative to more abundant transcripts at each of the six stages of bovine preimplantation development examined. The identification of so many translationally suppressed, abundant to moderately abundant, transcribed genes, i.e. mode 3 genes, was somewhat surprising. The transcripts of these genes were largely absent from the polysome fractions, were most abundant in the oocyte and two-cell stages, and diminished in number thereafter. A more detailed informatics analysis of these transcripts and an even more comprehensive time-course analysis seems warranted. One possibility is that the proteins encoded by these transcripts may be extremely stable or particularly efficient in their roles, so that low amounts of protein relative to mRNA are required for early development. Clearly, any interpretation of the roles of the genes within either of these groups based solely on the levels of their transcripts is bound to be incomplete. In conclusion, our study reveals unanticipated translational selectivity mechanisms operating on numerous genes across the genome. It identifies potentially important candidate regulators in embryonic programming that most likely have been overlooked in prior studies.

The analysis of genes in mode 2 (active, but modest translation of a selection of highly expressed mRNAs), i.e. those that would likely predominate in a bulk transcriptomic analysis, revealed a sequential progression of stage-specific gene networks accompanying development. The data are largely consistent with the sequential changes revealed in our previous analysis of co-expressed genes in bovine oocyte and preimplantation embryo transcriptomes (Jiang et al., 2014), but again reveal how transcriptomic data alone can be misleading and might overestimate the contribution of specific gene products to development. The transcripts that comprise mode 4 contribute weakly to the transcriptome except at the MII oocyte stage (Fig. 2), but appear to associate largely with monosomes and not be actively translated at the stages examined. Perhaps this association provides a mechanism wherein excess transcripts are not always translated but remain poised for future active translation. In other words, mode 4 mRNAs associated specifically with monosomes may constitute a novel but temporary storage state for transcripts.

Our analysis attempted to find whether there were genome-wide correlations between translational efficiency, which, for example, appears to be high in mode 1 genes and low in mode 3 and 4 genes, and certain transcript features. Consistent with the findings in mouse oocytes and embryos (Luong et al., 2020; Xiong et al., 2022), a high CPE density and length of 3′ UTR correlated with low TE. A complete annotation of the bovine functional genome will likely provide more insights into how such modes of translational selectivity are established.

The data also show that there are consistent translational similarities between the GV oocyte, the MII oocyte and the two-cell stage (Fig. 3A,C), but that there is a major translational perturbance evident at the eight-cell stage (Fig. 1B, Fig. 3A, Fig. 4A; Fig. S4), when the embryonic genome begins to contribute in a major way to the transcriptome. The transcripts identified in these early stages, i.e. GV oocyte to two-cell embryo were, as expected, mainly of maternal origin (Fig. S4A,B), but still fell within the four modes with different levels of TE. Prior to the eight-cell stage and also subsequently at the morula and blastocyst stages, translational dynamics were broadly correlated with the transcriptome. There was, however, a minor amount of transcriptional activity involving the embryonic genome at the two-cell stage that was reported (Graf et al., 2014; Jiang et al., 2014), and this appeared to correlate with high monosome occupancy by mRNA (Fig. 1C, Fig. 3A). The implications of this observation are unclear.

Transcripts encoding proteins involved in mitochondrial function, including ‘oxidation-reduction’, ‘electron transport chain’ and ‘mitochondrial translational initiation and elongation’, although not necessarily abundant, are efficiently selected for translation at all stages of development (Table 1), reflecting the essential role of mitochondria in generating energy to support oocyte and embryo development (Fragouli and Wells, 2015). Transcripts encoding enzymes involved in a wide array of metabolic pathways are also preferentially translated at all stages, again not an unsurprising observation (Botros et al., 2008; Bracewell-Milnes et al., 2017; Krisher and Prather, 2012; Nel-Themaat and Nagy, 2011; Redel et al., 2012; Singh and Sinclair, 2007; Vander Heiden et al., 2009). Why these mRNAs are so efficiently handled by the protein synthesis machinery remains unclear. However, a deeper understanding of the metabolic networks operating during these stages might facilitate the improvement of medium formulations for *in vitro* oocyte maturation and embryo culture, and allow the development of biomarker assays for assessing oocyte and embryo competence.

It should be recognized that the oocytes and embryos used in this study are products of *in vitro* protocols. Neither oocyte maturation nor embryo development occur as efficiently under these conditions as they do *in vivo*, although new formulations are constantly being tested to improve the procedures. There is concern, therefore, that *in vitro* procedures not only contribute to some degree of developmental failure (Zhu et al., 2021), but also cause alterations in the transcriptome (Gad et al., 2012; Kepkova et al., 2011; Rabaglino et al., 2021) and the translatome. Thus, the translational dynamic trajectory observed here *in vitro* might be somewhat different from that occurring *in vivo*. Nonetheless, *in vitro* fertilization and embryo *in vitro* culture are widely used in livestock species and in human *in vitro* fertilization programs. In particular, transfer of *in vitro*-produced bovine embryos is a successful commercial practice in the cattle industry and has already surpassed the numbers of pregnancies achieved from *in vivo*-derived embryo transfers (www.iets.org). Therefore, the data obtained from the standard *in vitro* system used in the present paper has direct relevance to current practice in the clinic and on the farm. Although not currently feasible because of cost considerations relating to the numbers of oocytes and embryos required, a comprehensive comparison of translational dynamics of *in vitro* embryos with their *in vivo* counterparts might be of considerable interest.

Several new methods, including Ribo-STAMP (Brannan et al., 2021), LiRibo-seq (Zhang et al., 2022), scRibo-seq (VanInsberghe et al., 2021), imaging-based SunTag (Dufourt et al., 2021) and RNA-fluorescence *in situ* hybridization and the puromycilation proximity ligation assay (RNA-puro-PLA) (Jansova et al., 2021), have recently opened avenues for understanding translational regulation with unprecedented cellular resolution. The main advantage of SunTag and RNA-puro-PLA, in particular, is to permit the localization and dynamics of mRNA translation to be observed at a single-molecule resolution. The development of the optimized SSP-profiling protocol described in the present study has enabled the characterization of the translational status of mRNAs bound to different kinds of ribosomes (free subunits, monosomes and polysomes) to be studied and has provided a more comprehensive picture of translational control during bovine early development than ever achieved previously. Combined with highly sensitive, high-throughput mass spectrometry to permit full proteomics analyses (Budnik et al., 2018; Gao et al., 2017), our technology should be capable of providing detailed insights into the relative contributions of transcription, translation and protein stability to the amounts of individual proteins in the developing embryo, as well as into detailed regulatory mechanisms at play.

In summary, our study has revealed a previously unappreciated level of complexity in genome-wide translational selectivity mechanisms associated with oocyte maturation and embryo development. In particular, the selective translation of non-abundant mRNAs for vital metabolic purposes throughout development, the stage-specific translational suppression of abundant to moderately abundant mRNAs, and the range of mRNAs associated with monosomes were particularly striking observations. Our work has filled a significant knowledge gap in the study of translational regulation over a period of rapid developmental change and provided an extensive database that can be mined for more detailed insights into bovine oocyte and preimplantation development.

## MATERIALS AND METHODS

### Bovine oocytes and *in vitro* embryo production

Germinal vesicle stage oocytes (GV oocytes) were collected as cumulus-oocyte complexes from follicles of 3-5 mm in diameter aspirated from slaughterhouse *Bos taurus* ovaries. BO-IVM medium (IVF Bioscience) was used for oocyte *in vitro* maturation. Maturation was conducted in four-well dishes for 22-23 h at 38.5°C with 6% CO_2_ to collect MII oocytes. Cumulus cells were completely removed and maturation was confirmed by light microscopy examination. Cryopreserved semen from a Holstein bull with proven fertility was diluted with BO-SemenPrep medium (IVF Bioscience) and added to drops containing cumulus-oocyte complexes (COCs) with a final concentration of 2×10^6^ spermatozoa/ml. Gametes were co-incubated in 6% CO_2_ in air at 38.5°C for 18 h. Embryos were then washed and cultured in BO-IVC medium (IVF Bioscience) at 38.5°C with 6% CO_2_. Different developmental stage embryos (two-cell, eight-cell, morula and blastocyst) were then evaluated under light microscopy and only Grade 1 embryos by the standards of the International Embryo Technology Society (https://www.iets.org) were selected for further study. Prior to oocyte and embryo collection, 100 μg/ml of cycloheximide (Sigma-Aldrich) was added into the culture for 10 min to stabilize and halt ribosomes on transcripts. Oocytes and embryos were then washed with D-PBS (Thermo Fisher Scientific) containing 1 mg/ml polyvinylpyrrolidone (Sigma-Aldrich) (PBS-PVP) and transferred into 50 μl droplets of 0.1% protease (QIAGEN) to remove the zona pellucida. Oocytes and embryos were rinsed three times in PBS-PVP and confirmed to be free of contaminating cells, and then snap frozen in minimal medium and stored at −80°C until polysome fractionation.

### Isolation of ribosome-bound mRNA

Approximately 100 oocytes (GV or MII oocyte) or embryos at different developmental stages (two-cell, eight-cell, morula and blastocyst) were combined with lysis buffer containing 10 mM HEPES (pH 7.5), 5 mM KCl, 5 mM MgCl_2_, 2 mM dithiothreitol, 1% Triton X-100, 100 μg/ml cycloheximide, complete EDTA-free protease inhibitor (Roche) and 40 U/ml RNase inhibitor (RNase-OUT, Invitrogen). Oocytes and embryos were disrupted by zirconium silica beads (Sigma-Aldrich) in the mixer mill apparatus MM301 (shake frequency 30, total time 45 s, Retsch). Lysates were cleaned by centrifugation in 10,000 ***g*** for 5 min at 4°C and the supernatants were loaded into 10-40% linear sucrose gradients containing 10 mM HEPES (pH 7.5), 100 mM KCl, 5 mM MgCl_2_, 2 mM dithiothreitol, 100 μg/ml cycloheximide, complete EDTA-free protease inhibitor and 5 U/ml RNase inhibitor. Ultracentrifugation was carried out with a SW55Ti rotor and Optima L-90 ultracentrifuge (Beckman Coulter) at 45,000 RPM (246,078 ***g***). Ribosome profiles were recorded by ISCO UV absorbance reader (Teledyne, ISCO). The overall quality of ribosome fractionation experiments was monitored by parallel analysis of a HEK293 cell sample. Ten equal fractions were then recovered and subjected to RNA isolation by Trizol reagent (Sigma-Aldrich).

### qRT-PCR analysis

The RNA profile from each fraction was tested by qRT-PCR analysis with 18S and 28S rRNA-specific primers to reconstruct a distribution of non-polysomal and polysomal RNA complexes in each profile (Masek et al., 2020). Briefly, 2 μl of RNA from each fraction were reverse-transcribed using 20 U of M-MuLV Reverse Transcriptase (Thermo Fisher Scientific) and 0.3 μg of random hexamer primers in a reaction volume of 20 μl. cDNA synthesis was performed at 25°C for 10 min and then in 37°C for 5 min, followed by incubation at 42°C for 1 h and subsequent inactivation at 70°C for 10 min. qRT-PCR experiments were performed using the LightCycler480 SYBR Green I Master mix (Roche) on a LightCycler480 (Roche). The 10 μl reactions were performed in triplicate. Each reaction contained 2 μl of cDNA and 500 nM gene-specific primers (the list of primers used are provided in Table S4). The amplification protocol was as follows: 95°C for 5 min; 44 cycles of 95°C for 10 s, 58°C for 15 s, 72°C for 15 s; followed by melting curve determination. For absolute qRT-PCR quantification, we created recombinant pCRTM4-Topo plasmids (Invitrogen) containing 18S and 28S ribosomal RNA PCR amplicons. The relative quantification mode was applied and the mean of 18S and 28S RNA levels was used for the normalization of each fractionation (Fig. S1).

As described above, the RNA was separated in a sucrose gradient solution based on the number of ribosomes bound to the RNA. The 18S and 28S ribosomal subunits are central components of the 40S and 60S ribosomal subunits, respectively. Fractions 1 and 2 contained primarily free RNA; as a result, the concentration of the 18S and 28S would be expected to be low in comparison with the other fractions. Then, based on density, we anticipated high 18S rRNA and low 28S rRNA in fractions with 40S small ribosomal subunits, and low 18S rRNA and high 28S rRNA in fractions with the 60S large subunits. Both would be present in the 80S monosomes and in polysomes, the sizes of which would be evident from their alternating increasing content of both rRNAs. Therefore, the quantification of the 18S and 28S rRNA provides direct information on the reliability of fraction collection (Masek et al., 2020).

### Library preparation and RNA-seq

The RNA-seq libraries were generated from individual fractions by using the Smart-seq2 v4 kit (Clontech) with minor modifications from the manufacturer's instructions. Briefly, individual cells were lysed and mRNA was captured and amplified with the Smart-seq2 v4 kit. After AMPure XP beads (Beckman) purification, the amplified RNAs were quality checked by using the High Sensitivity D5000 kit (Agilent Technologies). High-quality amplified RNAs were subject to library preparation (Nextera XT DNA Library Preparation Kit; Illumina) and multiplexed by Nextera XT Indexes (Illumina). The concentration of sequencing libraries was determined by using the Qubit dsDNA HS Assay Kit (Life Technologies) and KAPA Library Quantification Kits (KAPA Biosystems). The size of sequencing libraries was determined by the High Sensitivity D5000 Assay in a TapeStation 4200 system (Agilent). Pooled indexed libraries were then sequenced on the Illumina HiSeq X platform with 150-bp paired-end reads.

A pool of 20 oocytes or preimplantation embryos (*n*=3) selected from the same batch in each developmental stage used for ribosome profiling was used to profile transcriptomes by RNA-seq following the Smart-seq2 protocol as above described. In total, we sequenced 138 RNA-seq libraries (120 ribosome-bound mRNA libraries and 18 whole transcriptomes) and we generated approximately 40 million 150 bp paired-end reads per sample.

### RNA-seq data analysis

The Salmon tool (Patro et al., 2017) was applied to quantify the genome-wide gene expression profile from the raw sequencing data, by using the Ensembl bovine genome annotation (ARS-UCD1.2). Transcript per million reads (TPM) was used as the unit of mRNA level. The edgeR tool (Robinson et al., 2010) was applied to identify differentially expressed genes. The TMM algorithm implemented in the edgeR package was used to perform normalization of the read counts and estimation of the effective library sizes. Differential expression analysis was performed by the likelihood ratio test implemented in the edgeR package.

In this study, the fractions of free RNAs (F1 and F2) were excluded because of the discontinuity with the other fractions in the global expression pattern (Fig. 1B), and also because no ribosome-bound RNA was detected in these fractions by qRT-PCR analysis as described above. We anticipated that the largely free RNA (not attached to any ribosomes or proteins) in the F1 and F2 fractions might include microRNAs or non-coding RNAs, which play a significant function in early development based on recently studies (Ganesh et al., 2020; Hasuwa et al., 2021; Kataruka et al., 2020; Loubalova et al., 2021). The inadequate annotation of such RNAs in the bovine genome also limited the comprehensive characterizations in this study.

To understand the translational selectivity in each developmental stage, Spearman's rank correlation test was applied to compute the relationship between gene expression and consecutive ribosomal fractions (F3-F10). The genes with significant gradual increase or decrease in expression were retained for further analysis.

We also performed genome-wide correlation analysis between the transcripts that constituted the four different modes and had certain characteristic mRNA features. The transcripts with 5′ UTR or 3′ UTR length ≤100 nt were excluded when investigating 5′ UTRs and 3′ UTRs. The CPEs within 3′ UTRs were identified based on the motif sequences ‘TTTTAT’, ‘TTTTAAT’, ‘TTTTACT’, ‘TTTTCAT’, ‘TTTTAAAT’ and ‘TTTTAAGT’ (Luong et al., 2020; Xiong et al., 2022). We only retained the exact motif matches for the CPE number and density analysis.

We further performed analysis to determine whether or how maternal or embryonic transcripts are associated with different translational efficiency (four different modes). The maternal genes were defined as the genes strongly upregulated in both the GV and MII stages compared with the eight-cell, morula and blastocyst stages (FDR<0.05 and FC>4). The embryonic genes were the genes that were strongly upregulated in the eight-cell, morula and blastocyst stages relative to the GV and MII stages (FDR<0.05 and FC>4). The proportion of maternal/embryonic genes within each mode was computed as the number of maternal/embryonic genes in one given mode divided by the total number of genes in that mode.

All the conventional statistical analyses were performed using the R platform. The ‘cor.test’ function was used to perform Spearman's rank correlation test. A linear model controlling for fractionation was applied to prioritize the polysome-occupied genes with a gradual increase or decrease in expression across the developmental stages using the ‘lm’ function. If multiple testing needed to be accounted for, the ‘p.adjust’ function was applied for *P*-value correction. Principal component analysis on the genome-wide gene expression profile was performed by using the ‘dudi.pca’ function within the package ‘ade4’. All the heatmaps were plotted by the ‘heatmap.2’ function within the package ‘gplots’. The Gene Ontology and pathway analyses were performed by the David tool (Huang et al., 2009).

## Supplementary Material

10.1242/develop.200819_sup1 Supplementary information
