# Supplementary material for: High-resolution ribosome profiling reveals translational selectivity for transcripts in bovine preimplantation embryo development
Source: Development. 2022 Nov 3;149(21):dev200819. doi: 10.1242/dev.200819 (PMC9687001; doi:10.1242/dev.200819)
Supplement: Supplementary information [file develop-149-200819-s1.pdf]

Figure S1

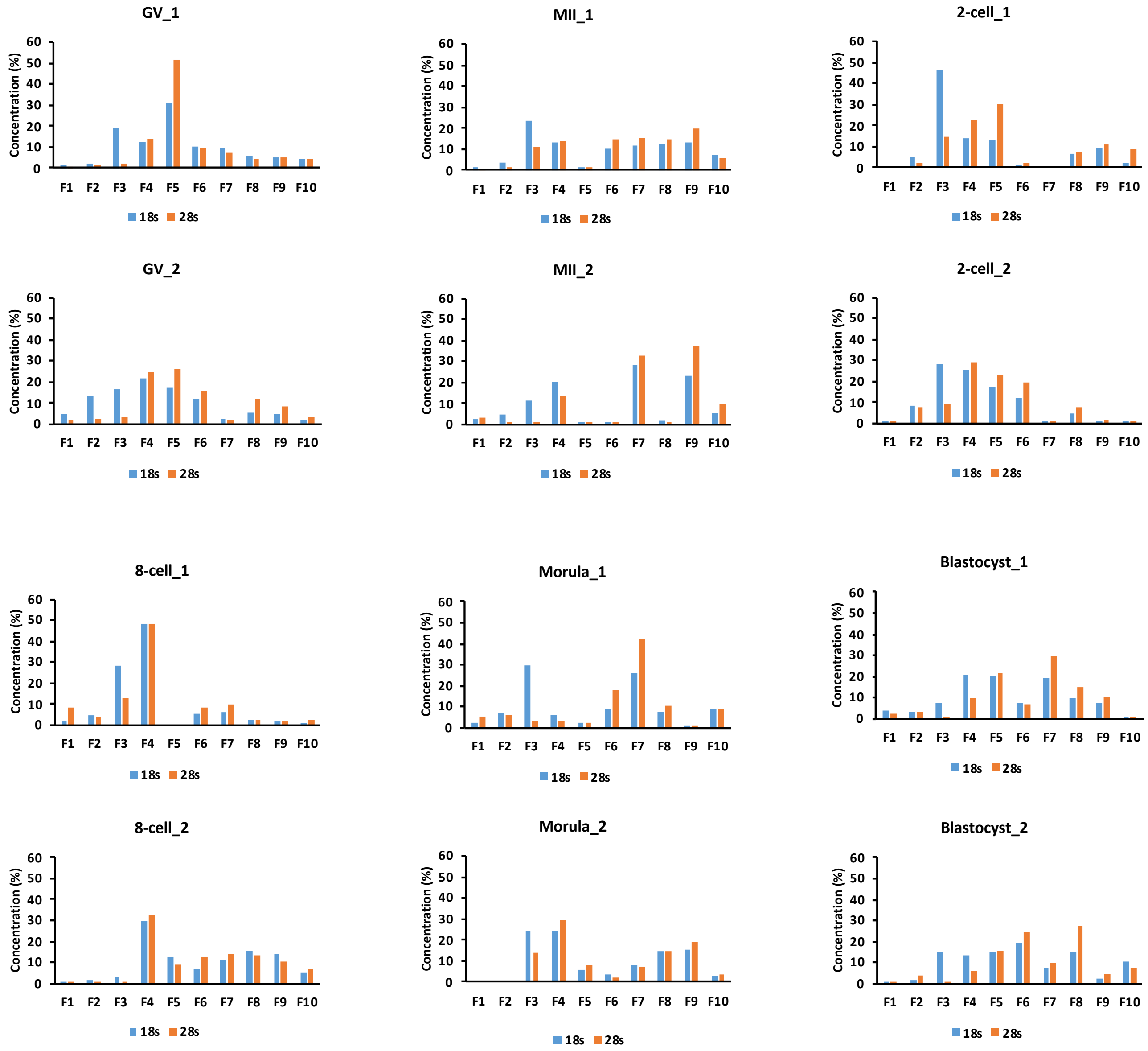

Fig. S1. qRT-PCR analysis of the distribution of 18S and 28S rRNA in each fraction of bovine oocyte and preimplantation development.

Development • Supplementary information

Figure S2

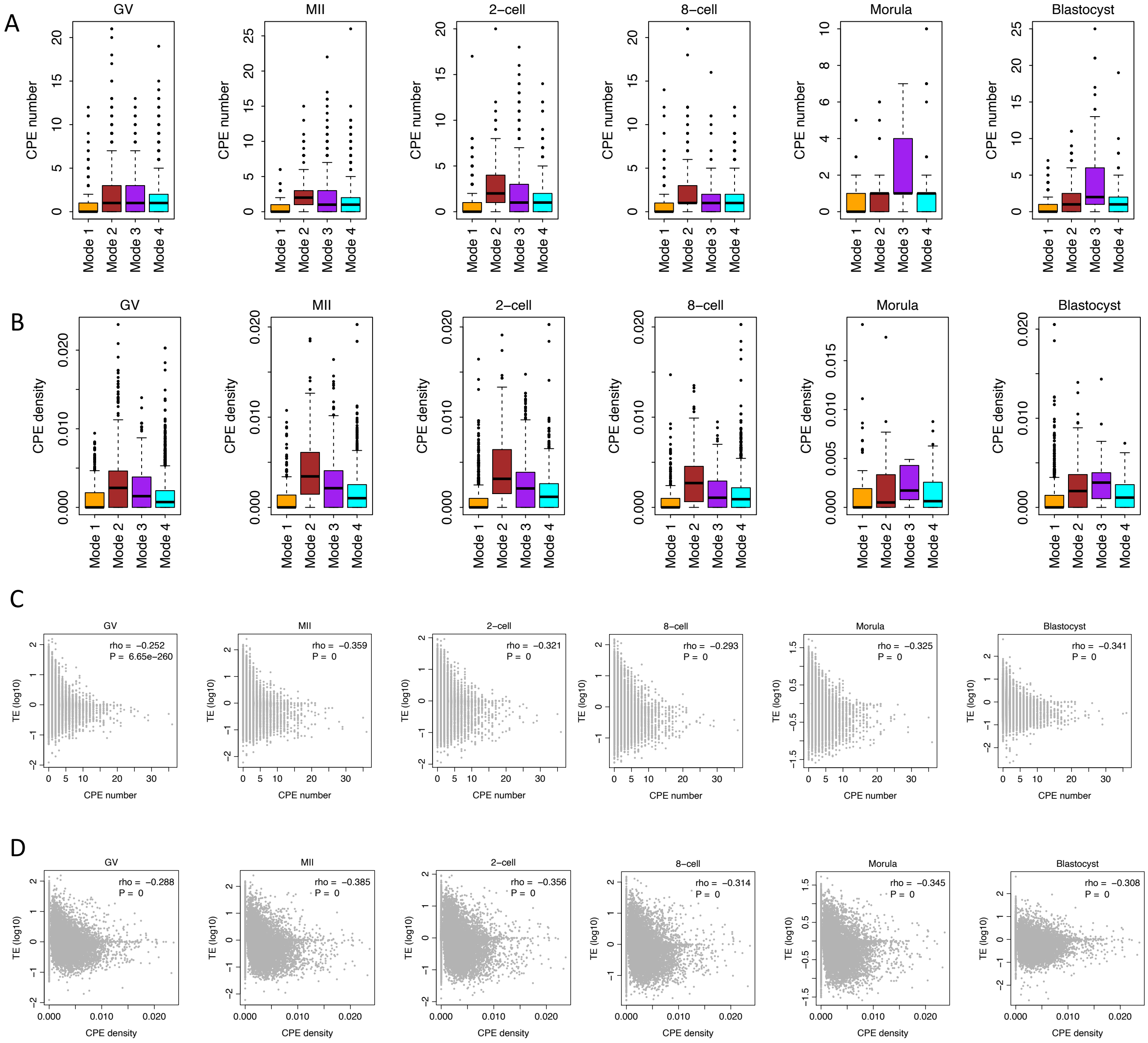

Development • Supplementary information

Fig. S2. Boxplots showing the CPE (Cytoplasmic polyadenylation elements) number (A) and density (B) of transcripts in identified four modes of translational selectivity across bovine oocyte and preimplantation development. Spearman correlations showing genome-wide relationship between translational efficiency [TE, (polysomeF8-F10 + 1) / (mRNA + 1)] and CPE number (C), and CPE density (D).

Figure S3

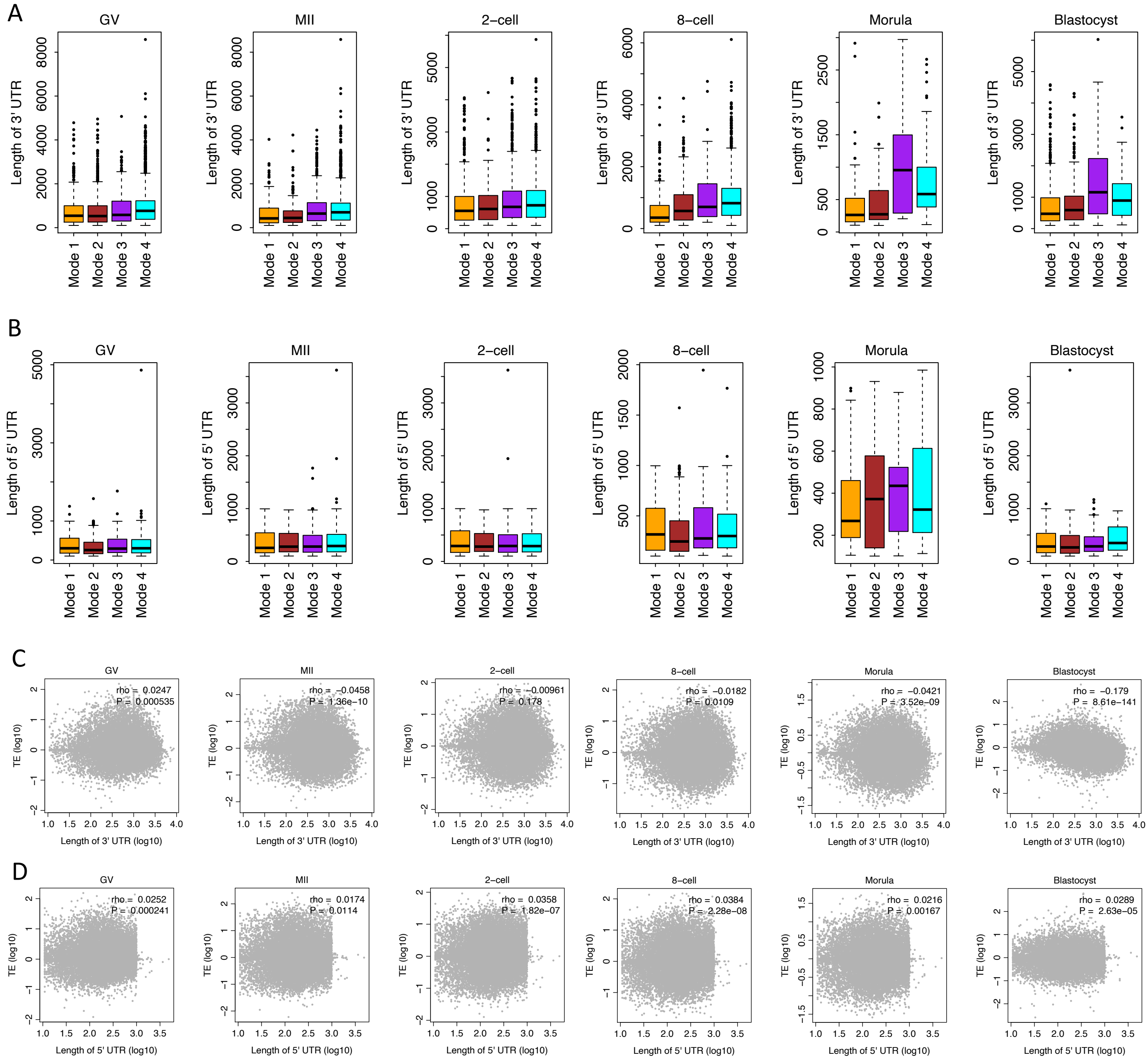

Development • Supplementary information

Fig. S3. Boxplots showing the 3' UTR (A) and 5' UTR (B) length of transcripts in identified four modes of translational selectivity across bovine oocyte and preimplantation development. Spearman correlations showing genome-wide relationship between translational efficiency [TE, (polysomeF8-F10 + 1) / (mRNA + 1)] and 3' UTR (C) and 5' UTR (D) length.

Development • Supplementary information

Figure S4

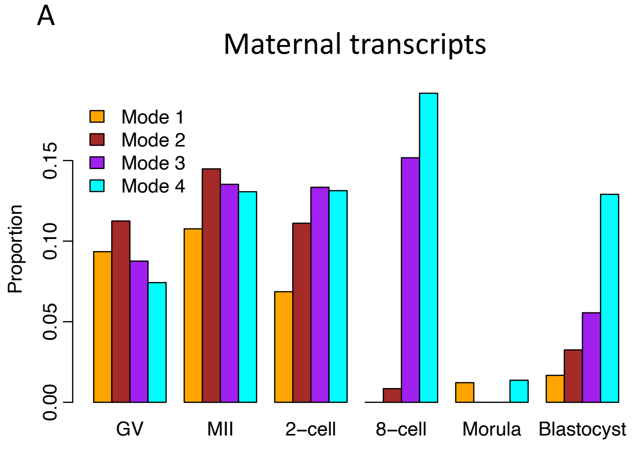

Fig. S4. The proportion of the identified maternal (A) or embryonic (B) transcripts in the four Modes of translational selectivity across bovine oocyte and preimplantation development.

Figure S5

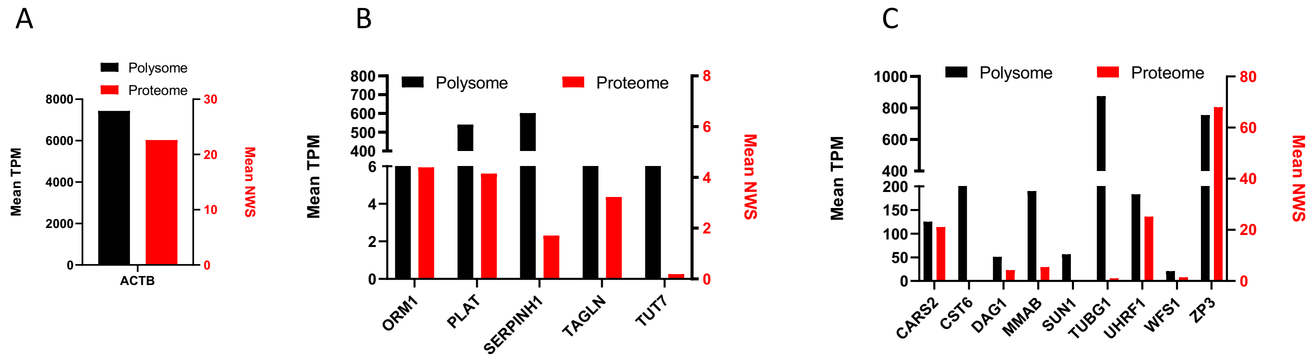

Fig. S5. Comparison of identified marked polysome bounded genes (Blue colored in Fig. 5 in this study) and proteomics data (Banliat et al., 2022) in bovine preimplantation embryos. A. Actin gene was used in this analysis to validate the protein mass spectrometry with RNA-seq data at 8-cell embryos. B. The expression of five highly translated genes (ORM1, PLAT, SERPINH1, TAGLN, and TUT7) in 8-cell embryos and their abundant protein products at the same stage. C. The expression levels of identified marked polysome bounded genes (CARS2, CST6, DAG1, MMAB, SUN1, TUBG1, UHRF1, WFS1 and ZP3) from other developmental stages are compared with respective protein abundance at matched developmental stages.

Development • Supplementary information

Table S1. A complete list of genes from identified four modes of translational selectivity across bovine oocyte and preimplantation development. In each of developmental stage, spreadsheet lists genes from four different modes as specific by 1, 2, 3, and 4, respectively.

Click here to download Table S1

Table S2. A list of 103 genes that are identified to be down-regulated their expression, while up-regulated their actual translations in MII compared to GV oocytes (FDR < 0.05 and FC > 2).

Click here to download Table S2

Table S3. A list of 65 genes that are identified to be up-regulated their expression, while down-regulated their actual translations in during 2- to 8-cell transition (FDR < 0.05 and FC > 2).

Click here to download Table S3

Table S4. Primers for qPCR analysis of 18s and 28s rRNA in the RNA from each fraction of bovine oocyte and preimplantation development.

Click here to download Table S4

Development • Supplementary information
