# Supplementary material for: High-resolution ribosome profiling reveals translational selectivity for transcripts in bovine preimplantation embryo development
Source: Development. 2022 Nov 3;149(21):dev200819. doi: 10.1242/dev.200819 (PMC9687001; doi:10.1242/dev.200819)
Supplement: Supplementary file 1 [file develop-149-200819_review_history.pdf]

High-resolution Ribosome Profiling Reveals Translational Selectivity for Transcripts in Bovine Preimplantation Embryo Development

Linkai Zhu, Tong Zhou, Rajan Iyyappan, Hao Ming, Michal Dvoran, Yinjuan Wang, Qi Chen, R. Michael Roberts, Andrej Susor and Zongliang Jiang

DOI: 10.1242/dev.200819

Editor: Samantha Morris

Review timeline

Original submission: 5 April 2022
Editorial decision: 16 June 2022
First revision received: 6 September 2022
Accepted: 26 September 2022

Original submission

First decision letter

MS ID#: DEVELOP/2022/200819

MS TITLE: High-resolution Ribosome Profiling Reveals Translational Selectivity for Transcripts in Bovine Preimplantation Embryo Development

AUTHORS: Linkai Zhu, Tong Zhou, Rajan Iyyappan, Hao Ming, Yinjuan Wang, Michal Dvoran, Qi Chen, R. Michael Roberts, Andrej Susor, and Zongliang Jiang

I have now received all the referees' reports on the above manuscript, and have reached a decision. The referees' comments are appended below, or you can access them online: please go to BenchPress and click on the 'Manuscripts with Decisions' queue in the Author Area.

As you will see, the referees express considerable interest in your work, but have some significant criticisms and recommend a substantial revision of your manuscript before we can consider publication. If you are able to revise the manuscript along the lines suggested, which may involve further experiments, I will be happy receive a revised version of the manuscript. Your revised paper will be re-reviewed by one or more of the original referees, and acceptance of your manuscript will depend on your addressing satisfactorily the reviewers' major concerns. Please also note that Development will normally permit only one round of major revision. If it would be helpful, you are welcome to contact us to discuss your revision in greater detail. Please send us a point-by-point response indicating your plans for addressing the referee’s comments, and we will look over this and provide further guidance.

Please attend to all of the reviewers' comments and ensure that you clearly highlight all changes made in the revised manuscript. Please avoid using 'Tracked changes' in Word files as these are lost in PDF conversion. I should be grateful if you would also provide a point-by-point response detailing how you have dealt with the points raised by the reviewers in the 'Response to Reviewers' box. If you do not agree with any of their criticisms or suggestions please explain clearly why this is so.

© 2022. Published by The Company of Biologists under the terms of the Creative Commons Attribution License (https://creativecommons.org/licenses/by/4.0/).

Reviewer 1

Advance summary and potential significance to field

Main Comments: The authors performed the transcriptome, polysome- and non-polysome-bound RNA profiles of bovine oocytes (GV and MII stage) and early embryos at 2-, 8-cell, morula, and blastocyst stage, and revealed four modes of translational selectivity. They found that the translational dynamics largely reflects transcriptional profiles in oocytes and 2-cell embryos, but observed marked shift in translational control in 8-cell embryos associated with the main phase of embryonic genome activation; transcription and translation become better synchronized in morulae and blastocysts. The reviewer suggests the MS is interesting, the profiling is of importance to reveal gene expression patterns in bovine preimplantation development, which can provide useful information for cattle reproduction and breeding, as well as a model for human reproduction.

Comments for the author

However, it is important to screen out the mark genes at different development stages such as 2-celled, 8-celled, morula and blastocyst stage, indicating their function in directing competent development of early embryos. These mark genes or their proteins can be used to verify the quality of embryos, especially for post-implantation development.

Minor comments:

1. Supplementary table 3. A list of 65 genes can be presented in the main text to indicate which can be candidate genes for competent development.
2. Reference format need to be carefully checked, some references are not complete, such as Line 580,601,671,754. Some out-dates references may need to delete.

Reviewer 2

Advance summary and potential significance to field

This study uses an elegant ribosome profiling approach to estimate translation efficiencies (TEs) of mRNAs during bovine oocyte development and in early embryos (2-cell, 4-cell, 8-cell, morula, blastocyst). Thereby the study provides for the first time a comprehensive overview of the translational dynamics during bovine preimplantation development. Another novel aspect is the differentiation between polysome- and non-polysome-bound mRNAs. Based on the comparison of global mRNA transcriptome profiles and ribosome-bound mRNA profiles, the authors arbitrarily define 4 categories of transcripts based on their abundances and TEs: i) low abundant transcripts with high TE; ii) mRNAs with high abundance but moderate TE; iii) transcripts with medium to high abundance but low TE; and iv) transcripts with different abundances associated with monosomes. These categories are displayed over all developmental stages, and standard ontology and functional annotation clustering tools are used to speculate on the functions of the genes in the different categories. The discussion of the obtained functional categories is reasonable.

Comments for the author

What is unfortunately missing is any mechanism that explains these observations.
For instance, are there any peculiarities e.g. in the 5’- or 3’-UTR sequences or in polyadenylation of the category i) genes that can explain their high translational efficiency? A recent paper on translational dynamics during maturation of mouse oocytes may provide some clues how these mechanistic aspects can be addressed (Luong et al., Nucleic Acids Research 48, 3257–3276, 2020). Another question is whether the observed patterns are associated with the rates of decay of maternal transcripts vs. production of embryonic transcripts which may have different TEs? This reviewer fully acknowledges the enormous effort that has been made to conduct the study, but without any mechanistic explanation the data remains – though comprehensive – descriptive.

© 2022. Published by The Company of Biologists under the terms of the Creative Commons Attribution License (https://creativecommons.org/licenses/by/4.0/).

Minor comment:
Lines 54 ff: there are several proteome studies of bovine oocytes and embryos e.g.:
Banliat et al., Dynamic Changes in the Proteome of Early Bovine Embryos Developed In Vivo. Front Cell Dev Biol. 2022 Mar 21;10:863700. doi: 10.3389/fcell.2022.863700. PMID: 35386205; PMCID: PMC8979002.
Banliat et al., Use of MALDI-TOF mass spectrometry to explore the peptidome and proteome of in-vitro produced bovine embryos pre-exposed to oviduct fluid.
Reprod Biol. 2021 Dec;21(4):100545. doi: 10.1016/j.repbio.2021.100545. Epub 2021 Aug 19. PMID: 34419706.
Marei et al., Proteomic changes in oocytes after in vitro maturation in lipotoxic conditions are different from those in cumulus cells. Sci Rep. 2019 Mar 6;9(1):3673. doi: 10.1038/s41598-019-40122-7. PMID: 30842615; PMCID:
PMC6403224.
Demant et al., Proteome analysis of early lineage specification in bovine embryos. Proteomics. 2015 Feb;15(4):688-701. doi: 10.1002/pmic.201400251. Epub 2014 Oct 9. PMID: 25143135.
Deutsch et al., Stage-specific proteome signatures in early bovine embryo development. J Proteome Res. 2014 Oct 3;13(10):4363-76. doi: 10.1021/pr500550t.
Epub 2014 Sep 10. PMID: 25102770.

First revision

Author response to reviewers' comments

Point-to-Point Response

We would like to thank both Reviewers for their valuable comments (in **bold black color**), which have helped improve the manuscript significantly. We have addressed the points (in blue color) raised by each of the Reviewers with new data, as described below.

Reviewer 1 Advance Summary and Potential Significance to Field:

Main Comments: The authors performed the transcriptome, polysome- and non-polysome-bound RNA profiles of bovine oocytes (GV and MII stage) and early embryos at 2-, 8-cell, morula, and blastocyst stage, and revealed four modes of translational selectivity. They found that the translational dynamics largely reflects transcriptional profiles in oocytes and 2-cell embryos, but observed marked shift in translational control in 8-cell embryos associated with the main phase of embryonic genome activation; transcription and translation become better synchronized in morulae and blastocysts. The reviewer suggests the MS is interesting, the profiling is of importance to reveal gene expression patterns in bovine preimplantation development, which can provide useful information for cattle reproduction and breeding, as well as a model for human reproduction.

We would like to thank Reviewer 1 for the very positive comments.

Reviewer 1 Comments for the Author:
However, it is important to screen out the mark genes at different development stages such as 2- celled, 8-celled, morula and blastocyst stage, indicating their function in directing competent development of early embryos. These mark genes or their proteins can be used to verify the quality of embryos, especially for post-implantation development.

Thank you for the comments. We agree with the Reviewer that a brief list of marker genes from various developmental stages is necessary. The same analysis and data are provided in the original supplementary **Figure S2**, but we have now moved this figure into the main text, **Figure 5C**. Those genes identified to be highly translated and/or transcribed at one particular stage but have low expression at other stages, indicating their specific regulatory function associated with a particular transition and in directing embryo competence.

© 2022. Published by The Company of Biologists under the terms of the Creative Commons Attribution License (https://creativecommons.org/licenses/by/4.0/).

Figure 5C

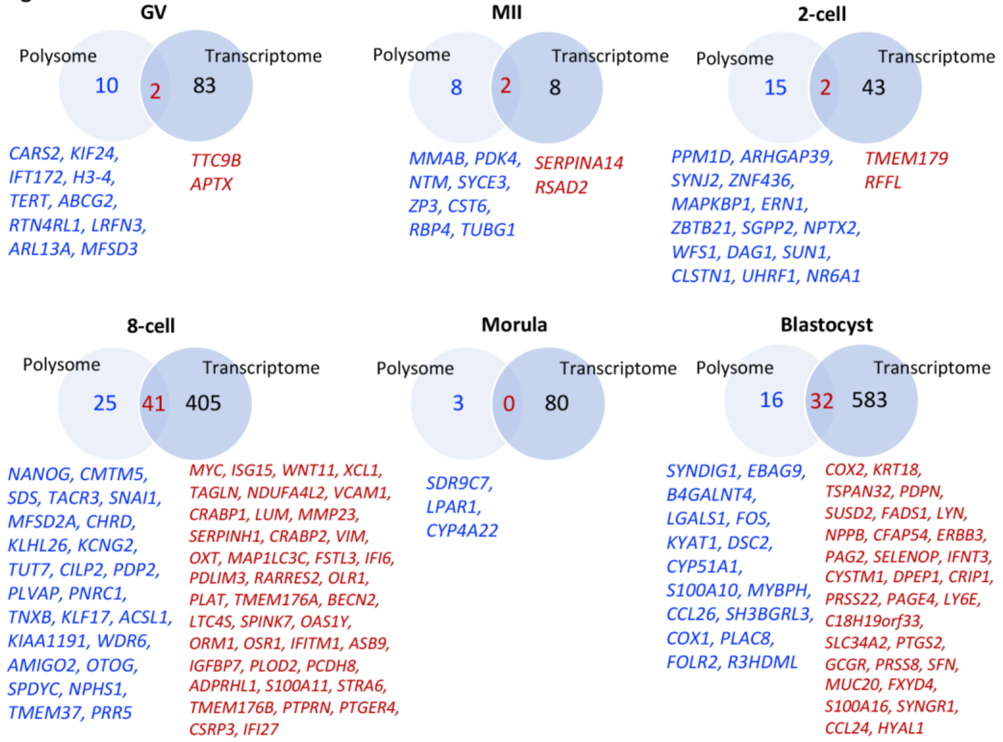

We have also attempted to validate these identified genes. Unfortunately, many of the antibodies did not have the necessary cross-species reactivity against bovine antigens. Instead, we took the advantage of an available bovine embryo proteomics dataset published recently (as suggested by Reviewer 2) as well, and were able to validate five genes in the 8-cell embryos and an additional nine genes across other stages. These new results are presented on Lines 313-320, and in a new figure (Figure S5).

Figure S5

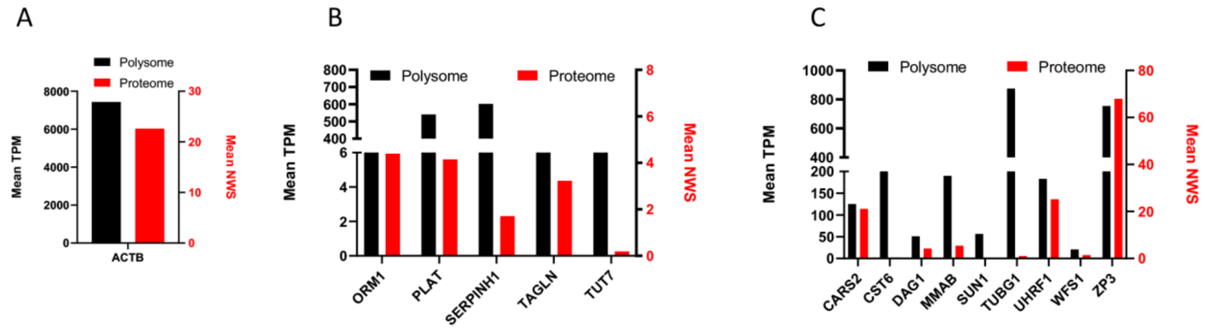

Similarly, we have also prioritized 90 genes that have the most dynamic translational selectivity across development (Figure 4B) and shortlisted seven top ranked genes (Figure 4C). Many of them play roles in metabolic networks and as epigenetic modifiers. As suggested, these select translated genes in bovine early embryo development might allow the development of biomarker assays for assessing oocyte and embryo competence.

With the combination of the original data mentioned above and the new validation data (Figure S5), we believe that we have identified a vast database of potential candidate/marker genes for bovine oocyte and embryo competence. We hope the reviewer can appreciate this.

© 2022. Published by The Company of Biologists under the terms of the Creative Commons Attribution License (https://creativecommons.org/licenses/by/4.0/).

Minor comments:
1. Supplementary table 3. A list of 65 genes can be presented in the main text to indicate which can be candidate genes for competent development.

We appreciate Reviewer's suggestion to highlight the potential candidate genes (highly translated ones) for oocyte and embryo competence.

Supplementary table 2 and 3 are the lists genes that had decreased transcription but an up-regulation of translation (gold dots), or increased transcription but decreased translation (blue dots) at oocyte maturation (Table S2), or major EGA (Table S3) respectively, their enriched GO terms were presented in Figure 5B. The purpose of this analysis is to provide specific examples of genes showing discordance between transcription and translation.

As part of responses to your major comments above, we have identified important marker genes and listed in the main text. We have also moved an original supplementary **Figure S2** into the main figure, new **Figure 5C**. We believe without misleading, those data together with new validation data (Figure S5) should be given priority when considering further function studies for oocyte and embryo competence.

2. Reference format need to be carefully checked, some references are not complete, such as Line 580,601,671,754. Some out-dates references may need to delete.

Accepted. We have checked, updated all references using EndNote X9, and removed the outdated ones.

Reviewer 2 Advance Summary and Potential Significance to Field:
This study uses an elegant ribosome profiling approach to estimate translation efficiencies (TEs) of mRNAs during bovine oocyte development and in early embryos (2-cell, 4-cell, 8-cell, morula, blastocyst). Thereby the study provides for the first time a comprehensive overview of the translational dynamics during bovine preimplantation development. Another novel aspect is the differentiation between polysome- and non-polysome-bound mRNAs. Based on the comparison of global mRNA transcriptome profiles and ribosome-bound mRNA profiles, the authors arbitrarily define 4 categories of transcripts based on their abundances and TEs: i) low abundant transcripts with high TE; ii) mRNAs with high abundance but moderate TE; iii) transcripts with medium to high abundance but low TE; and iv) transcripts with different abundances associated with monosomes. These categories are displayed over all developmental stages, and standard ontology and functional annotation clustering tools are used to speculate on the functions of the genes in the different categories. The discussion of the obtained functional categories is reasonable.

We would like to thank Reviewer 2 for highlighting the strength of our manuscript.

Reviewer 2 Comments for the Author:
What is unfortunately missing is any mechanism that explains these observations. For instance, are there any peculiarities e.g., in the 5'- or 3'-UTR sequences or in polyadenylation of the category i) genes that can explain their high translational efficiency? A recent paper on translational dynamics during maturation of mouse oocytes may provide some clues how these mechanistic aspects can be addressed (Luong et al., Nucleic Acids Research 48, 3257-3276, 2020). Another question is whether the observed patterns are associated with the rates of decay of maternal transcripts vs. production of embryonic transcripts which may have different TEs? This reviewer fully acknowledges the enormous effort that has been made to conduct the study, but without any mechanistic explanation the data remains - though comprehensive - descriptive.

First of all, we acknowledge that our study is a comprehensive description of the genome-wide translational dynamics and translational selectivity during bovine oocyte and pre-implantation development. On the other hand, we would like to emphasize that:

© 2022. Published by The Company of Biologists under the terms of the Creative Commons Attribution License (https://creativecommons.org/licenses/by/4.0/).

(1). Our methodology (explore transcripts associated with monosomes and different sizes of polysomes) represents a significant advance over the traditional ribosome profiling approaches that have been used previously including the recently published mouse studies which appeared during the review of our study (Zhang et al., 2022, Science Advances; Xiong et al., 2022, Nature Cell Biology), which generally ignores how specific mRNAs are preferentially selected for translation.

(2). One of the novel findings from our study is that it has revealed four modes of translational selectivity for transcripts in oocytes and embryos. None of this information could be readily inferred from transcriptomic data alone; nor from ribosome profiling. In this sense, our study is innovative and certainly contributes to our understanding of oocyte maturation and early embryo development, specifically, identification of subclass of mRNAs differentially utilized post-transcriptionally during different stages of bovine embryonic development.

(3). Our work has filled a significant knowledge gap in the study of translational regulation as it pertains to bovine oocyte and early embryo development, and has provided an extensive data base that can be mined for more detailed insights into the entire process from egg to blastocyst.

Actually, the four modes translational selectivity provide another way of describing translational efficiency (TE), with the highest efficiency in Mode 1 transcripts, followed by Modes 2 and 4, with the least efficient in Mode 3.

We thank Reviewer 2 for prompting us to investigate translational efficiency in more depth and perform the suggested new analysis to understand how such modes of translational selectivity might be established. Our findings are provided on Lines 182-208, Lines 385-390, Figure S2, Figure S3, and Figure S4:

(1). First, the cytoplasmic polyadenylation element (CPE) has been implicated in translational regulation. Therefore, we performed a genome-wide correlation between the identified four modes of transcripts and various mRNA features. We observed transcripts in Mode 1 [highest translational efficiency (TE), polysome/mRNA] had the lowest CPE number and density, whereas transcripts in Mode 2 (moderate TE) and Mode 3 (lowest TE) demonstrated higher CPE number and density than Mode 1 both before (Figure S2A) and after EGA stages (Figure S2B). When TE was compared with the CPE number and density on all detected transcripts, we confirmed that these values were negatively correlated (Figure S2C and S2D). The decreased in TE in the progression from Mode 1 through Mode 4 was also accompanied by an increased length of 3' UTR, but not 5' UTR of the transcripts (Figure S3A and S3B) across all stages, and for all transcripts identified, TE was in general negatively correlated with 3' UTR length and positively correlated with 5' URT length. It should be noted, however, that these correlations were quite weak (Figure S2C and S2D). Together, these data reveal a role of CPE, and possibly length of 3' URT and 5' UTR for translation regulation in bovine early embryonic development.

(2). Second, we calculated the proportion of maternal and embryonic transcripts in the four Modes across developmental stages. The data showed that there are consistent translational similarities between the GV oocyte, the MII oocyte, and the 2-cell stages (Figure 3A & C), but that there is a major translational perturbance evident at the 8-cell stage (Fig.1B, Fig. 3A, Fig. 4A. Figure S4), when the embryonic genome begins to contribute in a major way to the transcriptome. The transcripts identified in these early stages, i.e., GV oocyte to 2-cell embryo are, as expected, mainly of maternal origin (Figures S4A & S4B), but still fall into the four modes with different levels of TE.

(3). Finally, we would like to remind the Reviewer that the bovine genome and functional genome annotation are not as complete as for mouse and humans. Therefore, we could not obtain meaningful poly-A information (length, etc.) for analysis in this study as suggested.

Together, we have provided additional analysis as a mechanistic explanation of the observed four modes of translational selectivity.

© 2022. Published by The Company of Biologists under the terms of the Creative Commons Attribution License (https://creativecommons.org/licenses/by/4.0/).

Figure S2

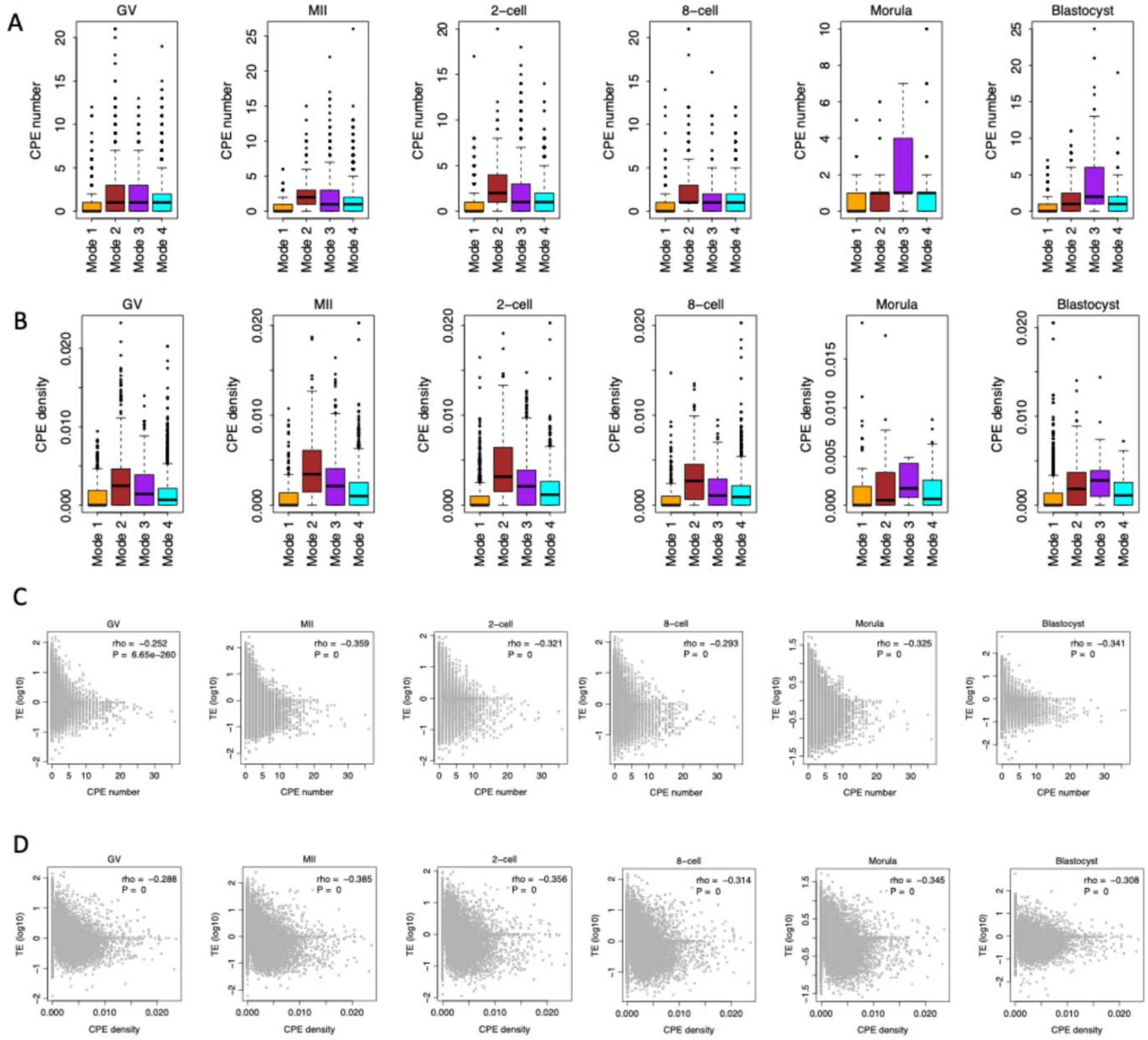

© 2022. Published by The Company of Biologists under the terms of the Creative Commons Attribution License (https://creativecommons.org/licenses/by/4.0/).

Figure S3

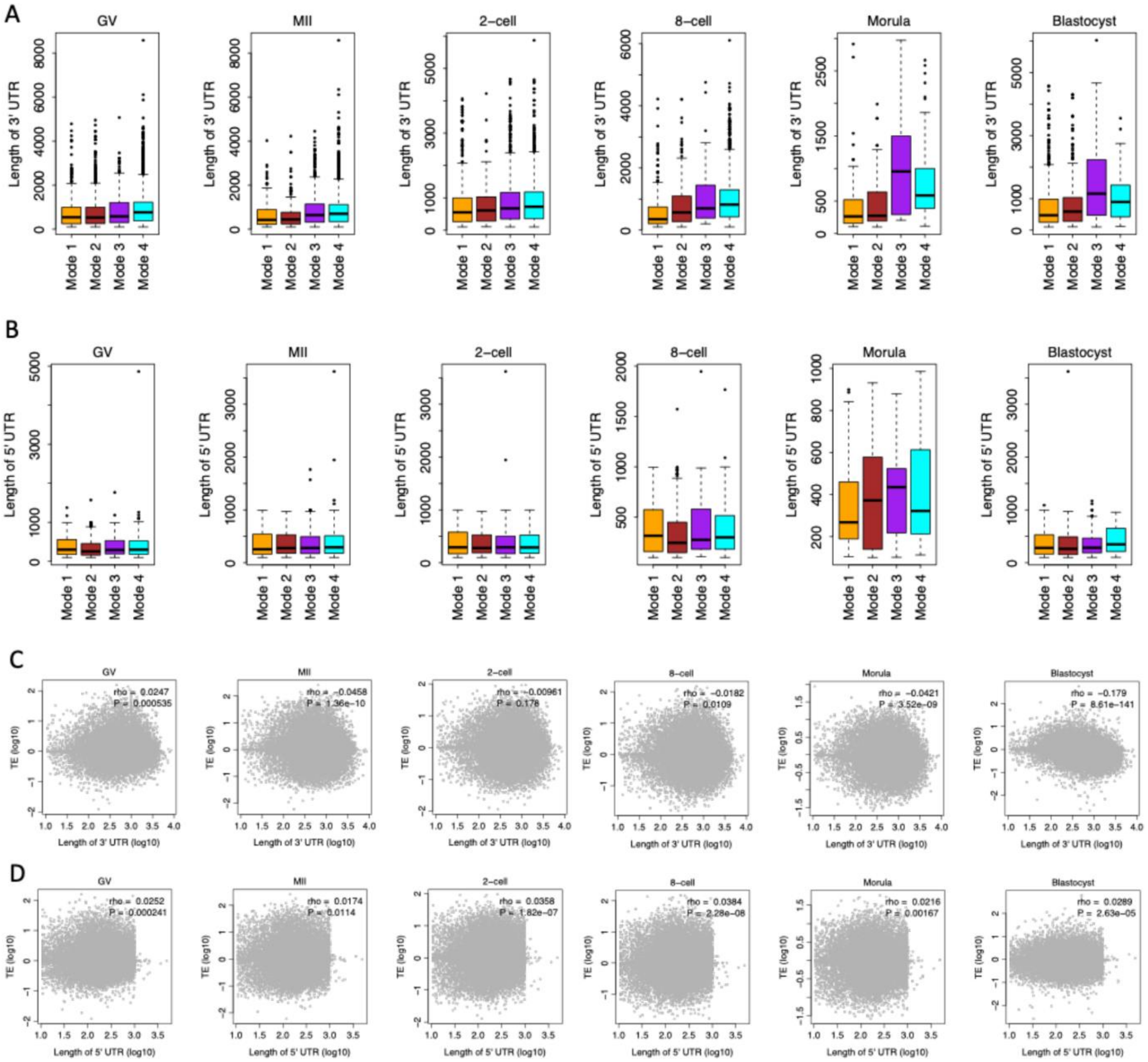

Figure S4

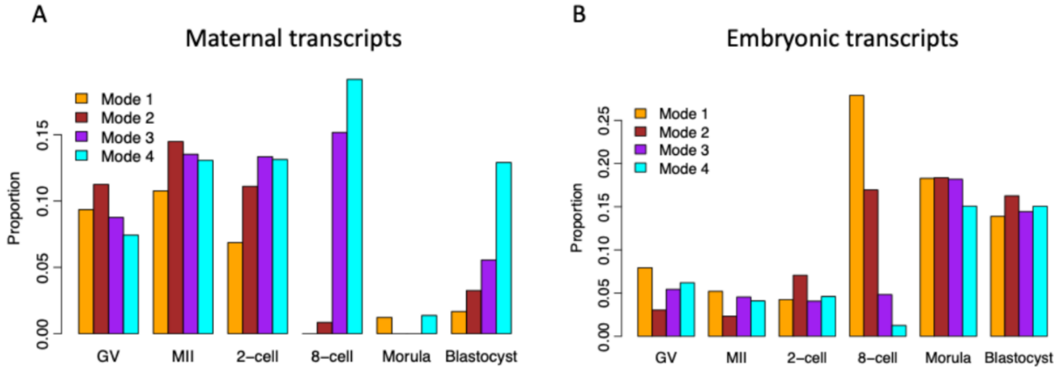

Minor comment:

Lines 54 ff: there are several proteome studies of bovine oocytes and embryos, e.g.:

- **Banliat et al., Dynamic Changes in the Proteome of Early Bovine Embryos Developed In Vivo. Front Cell Dev Biol. 2022 Mar 21;10:863700. doi: 0.3389/fcell.2022.863700. PMID: 35386205; PMCID: PMC8979002.**
- **Banliat et al., Use of MALDI-TOF mass spectrometry to explore the peptidome and proteome of in-vitro produced bovine embryos pre-exposed to oviduct fluid. Reprod Biol.**

© 2022. Published by The Company of Biologists under the terms of the Creative Commons Attribution License (https://creativecommons.org/licenses/by/4.0/).

2021 Dec;21(4):100545. Doi 10.1016/j.repbio.2021.100545. Epub 2021 Aug 19. PMID: 34419706.

- **Marei et al., Proteomic changes in oocytes after in vitro maturation in lipotoxic conditions are different from those in cumulus cells. Sci Rep. 2019 Mar 6;9(1):3673. doi: 10.1038/s41598-019- 40122-7. PMID: 30842615; PMCID: PMC6403224.**
- **Demant et al., Proteome analysis of early lineage specification in bovine embryos. Proteomics. 2015 Feb;15(4):688-701. doi: 10.1002/pmic.201400251. Epub 2014 Oct 9. PMID: 25143135.**
- **Deutsch et al., Stage-specific proteome signatures in early bovine embryo development. J Proteome Res. 2014 Oct 3;13(10):4363-76. doi: 10.1021/pr500550t. Epub 2014 Sep 10. PMID: 25102770.**

Thank you for pointing out this. We have corrected our statement and cited these proteome studies in bovine oocytes and early embryos in Lines 51-52. Moreover, we have also utilized the bovine embryo proteomic data to validate the identified mark genes (specifically highly translated in particular embryonic stages, Figure S5).

Second decision letter

MS ID#: DEVELOP/2022/200819

MS TITLE: High-resolution Ribosome Profiling Reveals Translational Selectivity for Transcripts in Bovine Preimplantation Embryo Development

AUTHORS: Linkai Zhu, Tong Zhou, Rajan Iyyappan, Hao Ming, Michal Dvoran, Yinjuan Wang, Qi Chen, R. Michael Roberts, Andrej Susor, and Zongliang Jiang
ARTICLE TYPE: Research Article

I am happy to tell you that your manuscript has been accepted for publication in Development, pending our standard ethics checks.

Reviewer 1

Advance summary and potential significance to field

The authors demonstrated the important findings that a strong translational selection of low abundance transcripts involved in metabolic pathways and lysosome was found throughout bovine embryonic development. Notably, genes involved in mitochondrial function were prioritized for translation. We found that translation largely reflected transcription in oocytes and 2-cells, but observed marked shift in translational control in 8-cells associated with the main phase of embryonic genome activation.
Subsequently, transcription and translation become better synchronized in morulae and blastocysts. Together, these data reveal a unique spatiotemporal translational regulation that accompanies bovine preimplantation development.

Comments for the author

The authors addressed the reviewer's comments accordingly and revised the MS appropriately. The current version is acceptable.

© 2022. Published by The Company of Biologists under the terms of the Creative Commons Attribution License (https://creativecommons.org/licenses/by/4.0/).

Reviewer 2

Advance summary and potential significance to field
This study uses an elegant ribosome profiling approach to estimate translation efficiencies (TEs) of mRNAs during bovine oocyte development and in early embryos (2-cell, 4-cell, 8-cell, morula, blastocyst). Thereby the study provides for the first time a comprehensive overview of the translational dynamics during bovine preimplantation development. Another novel aspect is the differentiation between polysome- and non-polysome-bound mRNAs. Based on the comparison of global mRNA transcriptome profiles and ribosome-bound mRNA profiles, the authors arbitrarily define 4 categories of transcripts based on their abundances and TEs: i) low abundant transcripts with high TE; ii) mRNAs with high abundance but moderate TE; iii) transcripts with medium to high abundance but low TE; and iv) transcripts with different abundances associated with monosomes. In the revised version of the manuscript the authors additionally attempted to add mechanistic explanations for the formation of the 4 categories.

Comments for the author

Thank you for addressing my comments.

© 2022. Published by The Company of Biologists under the terms of the Creative Commons Attribution License (https://creativecommons.org/licenses/by/4.0/).
